# Temporal dynamics of gut microbiota and virome in preterm infants: insights from longitudinal metagenomic analysis

**DOI:** 10.3389/fcimb.2026.1598786

**Published:** 2026-03-09

**Authors:** Jinjie Huang, Xudong Yan, Qian Su, Huiying Tu, Zhangbin Yu, Dong Liu, Benqing Wu

**Affiliations:** 1 Jinan University, Guangzhou, China; 2 Department of Neonatology, Shenzhen People’s Hospital, The Second Clinical Medical College of Jinan University, The First Affiliated Hospital of Southern University of Science and Technology, Shenzhen, Guangdong, China; 3 Benqing Laboratory, Shenzhen Guangming District People’s Hospital, Shenzhen, Guangdong, China

**Keywords:** bacterial and viraldynamics, gut microbiota, infants, microbial colonization, preterm infants

## Abstract

**Introduction:**

Preterm infants exhibit heightened vulnerability to morbidity and mortality due to their underdeveloped immune systems and immature gastrointestinal tract. The gut microbiota plays a pivotal role in neonatal health, yet its establishment is influenced by multiple factors, including prematurity, antibiotic exposure, and feeding modalities. This study aimed to examine the interactions among gut bacteriophages, bacterial communities, and clinical variables in preterm infants to identify potential microbial biomarkers associated with health outcomes.

**Methods:**

We employed metagenomic shotgun sequencing and co-occurrence network analysis to characterize the virome and bacterial communities in 12 preterm neonates at 14 and 28 days post-birth. This approach enabled the identification of dynamic microbial colonization patterns and key bacterial species and bacteriophages associated with clinical parameters.

**Results:**

*Staphylococcus epidermidis* exhibited a significant decline over time, whereas *Enterococcus faecalis* and its associated bacteriophages showed progressive enrichment, becoming predominant by day 28. In contrast, the relative abundances of *Clostridioides difficile* and *Klebsiella pneumoniae* remained statistically stable between the two time points (14 vs. 28 days).

**Discussion:**

These findings suggest that microbial changes during the first month of life may reflect a combination of host developmental processes and external influences, such as antibiotic exposure or delivery mode. The observed microbial signatures provide preliminary insights into early gut microbiota and virome development in preterm infants. However, their functional relevance and long-term stability require confirmation in larger, well-powered longitudinal studies with denser temporal sampling. The enrichment of *Enterococcus faecalis* may indicate its opportunistic colonization potential in the preterm gut and warrants further investigation regarding its role in gut homeostasis and immune system maturation.

## Introduction

Preterm birth, defined as delivery prior to 37 completed weeks of gestation, constitutes approximately 11% of global live births (Bradley et al., 2025). These infants exhibit heightened vulnerability to health complications due to immature organ systems, particularly underdeveloped immune and gastrointestinal (GI) functions, which predispose them to infectious diseases and other morbidities. The gut microbiota, a complex ecosystem of symbiotic microorganisms, exerts critical regulatory roles in neonatal development through mechanisms including supporting nutrient absorption, immune function, and pathogen resistance (Rutayisire et al., 2016; Saturio et al., 2021). Despite the growing body of evidence that highlights the crucial role of the gut microbiota in the health and development of preterm infants (Beghetti et al., 2023; Groer et al., 2014), a significant gap remains in our understanding of the temporal evolution of both the bacterial and viral components within these microbial communities during the early postnatal period. The viral component, known as the virome, along with the bacterial microbiota, plays an essential role in modulating the gut microbial ecosystem (Zhang et al., 2023). Elucidating how these components evolve over time is critical for understanding the impact of preterm infants on health outcomes. Recent advancements in metagenomic sequencing technologies have revolutionized the field of microbiome research (Inglis and Edwards, 2022; Yen and Johnson, 2021). These cutting-edge techniques allow for the detailed and comprehensive profiling of microbial communities, providing unprecedented insights into their composition, structure, and dynamics (Morniroli et al., 2023; Chen et al., 2022). By leveraging these technologies, researchers can now explore the complex interactions between bacterial and viral populations within the gut microbiota, shedding light on how these interactions influence the development and health of preterm infants.

In this study, we conducted a comprehensive longitudinal investigation of the gut microbiota and virome in a cohort of 12 preterm infants, with samples systematically collected at 14 and 28 days post-birth. By leveraging advanced high-throughput sequencing technologies, we characterized the dynamic shifts in microbial colonization patterns during this critical early developmental window. This analysis aimed to identify key factors governing the assembly and evolutionary trajectories of microbial communities over time, thereby elucidating the underlying temporal dynamics. Our research sought to determine how perinatal and neonatal variables, such as antibiotic exposure, feeding modalities, and delivery mode, which modulate the compositional dynamics and succession patterns of bacterial and viral populations in the gut. The findings demonstrate potential to inform the development of precision interventions targeting the modulation of early-life gut microbiota. Through elucidating core determinants of microbial community assembly and stability, we can design strategies to foster a resilient, health-promoting microbiota, thereby optimizing clinical outcomes in this high-risk population. These interventions could encompass precision probiotic therapies, personalized nutritional approaches, and novel therapeutic modalities aimed at enhancing gut microbiota functionality and resilience in preterm infants. Finally, our work contributes to the broader objective of refining healthcare for preterm infants by deepening insights into microbial determinants of development. Through translational application of our findings, we aim to refine evidence-based practices and improve short-term and long-term health and developmental trajectories for these infants, who face elevated risks of complications due to premature birth.

## Results

### Data summary

We integrated 24 metagenomic and viral metagenomic sequencing datasets from preterm infants collected at two postnatal time points (12 samples at 14 days post-birth [Group A] and 12 samples at 28 days post-birth [Group B]; **Table 1**). Sequencing metadata for metagenomic and viral metagenomic datasets are summarized in **Supplementary Tables S1, S2**, respectively.

**Table 1:** .

Individuals situation	Sample	HJY	MJX	HJN	LJY	ZhKZ	ZhKR	LYY	ZhH	LZh	ZhZH	XZH	PYL
maternal characteristic	Mother_age(years)	30	36	36	39	28	28	33	34	37	26	33	30
	Antenatal corticosteroid	yes	yes	yes	yes	yes	yes	no	yes	yes	yes	yes	no
	Antenatal magnesium_sulfate	yes	yes	yes	yes	yes	yes	no	yes	yes	yes	yes	yes
	Antibiotic use within 24_hours prior_to_delivery	yes	yes	yes	yes	yes	yes	yes	yes	yes	yes	yes	yes
	chorioamnionitis	no	no	no	definite	subclinical	subclinical	definite	no	definite	definite	subclinical	definite
	Membrane breaking time(h)	0	0	0	29	8	8	0	0	0	0	0	11
	assisted reproduction	yes	no	yes	yes	no	no	yes	no	no	no	yes	no
	multicellular_condition	dichorionic diamniotic	no	dichorionic diamniotic	dichorionic diamniotic	monochorionic diamniotic	monochorionic diamniotic	no	no	no	dichorionic diamniotic	dichorionic diamniotic	no
	delivery_way	cesarean	cesarean	cesarean	cesarean	cesarean	cesarean	cesarean	cesarean	cesarean	vaginal	cesarean	cesarean
neonatal characteristics	Gender	Female	Female	Female	Female	Male	Male	Female	Male	Male	Male	Female	Female
	gestational_age(week)	30	27+1	30+3	30^+2^	30^+4^	30^+4^	27+6	30^+2^	29^+2^	24^+5^	27^+2^	31^+6^
	Birth_Weight(g)	1210	700	790	1440	1540	1420	950	1320	1350	660	1080	1010
	Admission_temperature(°C)	35	34.8	34.5	35.4	35.8	35.5	35.7	36.2	36.8	36.2	36.9	36.3
	Auxiliary_scores_for_Apgar_scoring_1min_score	5	2	2	3	5	5	4	1	3	3	2	3
	Auxiliary_scores_for_Apgar_scoring_5min_score	5	3	5	5	5	5	3	3	2	2	3	2
	Umbilical_artery_blood_PH	7.33	7.17	7.21	7.14	7.27	7.31	7	7	7.135	7	7	7
	Apgar_score_1min	10	8	7	9	10	9	4	8	8	4	10	10
	Apgar_score_5min	10	10	10	10	10	10	8	10	10	9	10	10
	neonatal_asphyxia	no	no	mild	no	no	no	mild	no	no	mild	no	no
	Smaller_than_gestational_age	no	no	yes	no	no	no	no	no	no	no	no	yes
	respiratory_support_days	10.29	71	36.92	20	15.25	7.54	37.38	21	22.67	67.42	38.23	3.71
	Number_of_days_of_intubation	48	59	60	32	26	31	69	3	44	0	42	21
	umbilical_vein_cannulation	no	no	no	no	no	no	yes	yes	yes	yes	yes	no
	Duration_of_microfeeding	2	6	10	3	2	2	14	5	6	10	3	2
	Initiation time of oral feeding_days post-birth	31.0194	56.1333	60.9514	22.6021	19.6722	28.5389	56.4146	30.0097	0.06181	79.1444	46.2049	27.716
	Type_of_initiation_of_oral_feeding	formula milk	formula milk	formula milk	formula milk	formula milk	formula milk	formula milk	formula milk	formula milk	mother's milk	deeply hydrolyzed formula milk	mother's milk
	Types_of_antibiotics_after_birth_4weeks	2	4	2	3	2	1	1	2	2	4	1	1
	Number_of_days_on_antibiotics_after_birth_4weeks	13	16	11	14	15	12	26	13	9	24	7	4
	Frequency of defecation_in_the first three days	5	2	2	3	2	2	2	6	3	1	2	12
neonatal major morbidity and discharge outcome	BPD	no	yes	yes	no	no	no	no	no	no	yes	yes	no
	septicaemia	no	no	no	yes	yes	no	no	no	no	no	no	no
	ROP	yes	yes	yes	no	yes	yes	yes	no	no	yes	yes	no
	IVH	no	no	no	no	no	no	yes	no	no	no	no	yes
	NEC	no	no	no	no	no	no	no	yes	no	no	no	no
	Survival without major comorbidities	yes	yes	yes	yes	yes	yes	no	yes	yes	no	yes	yes
	outcome	Survival	Survival	Survival	Survival	Survival	Survival	Survival	Survival	Survival	Survival	Survival	Survival

After removal of low-quality reads (see Methods), we obtained a total of 4.1 billion high-quality reads, with an average retention rate of 97.50% for metagenomic data and 89.52% for viral metagenomic data. Potential human DNA contamination was controlled by mapping reads to the human reference genome and discarding host-derived sequences. Following an additional round of host-filtration with BMTagger (Rotmistrovsky and Agarwala, 2011), the metagenomic datasets retained 1.7 billion host-clean reads (average 74 million reads per sample), whereas viral metagenomic datasets yielded 240 million host-clean reads (average 1 million reads per sample). Detailed statistics of host sequence removal for both datasets are provided in **Supplementary Table S3**.

Each sample was assembled individually to generate sample-specific contigs (see Methods). Assembly statistics for viral metagenomes, including contig number, total assembly length, N50, and maximum contig size, are summarized in **Supplementary Table S4**.

### Analysis of microbial community structure

**Figure 1:**
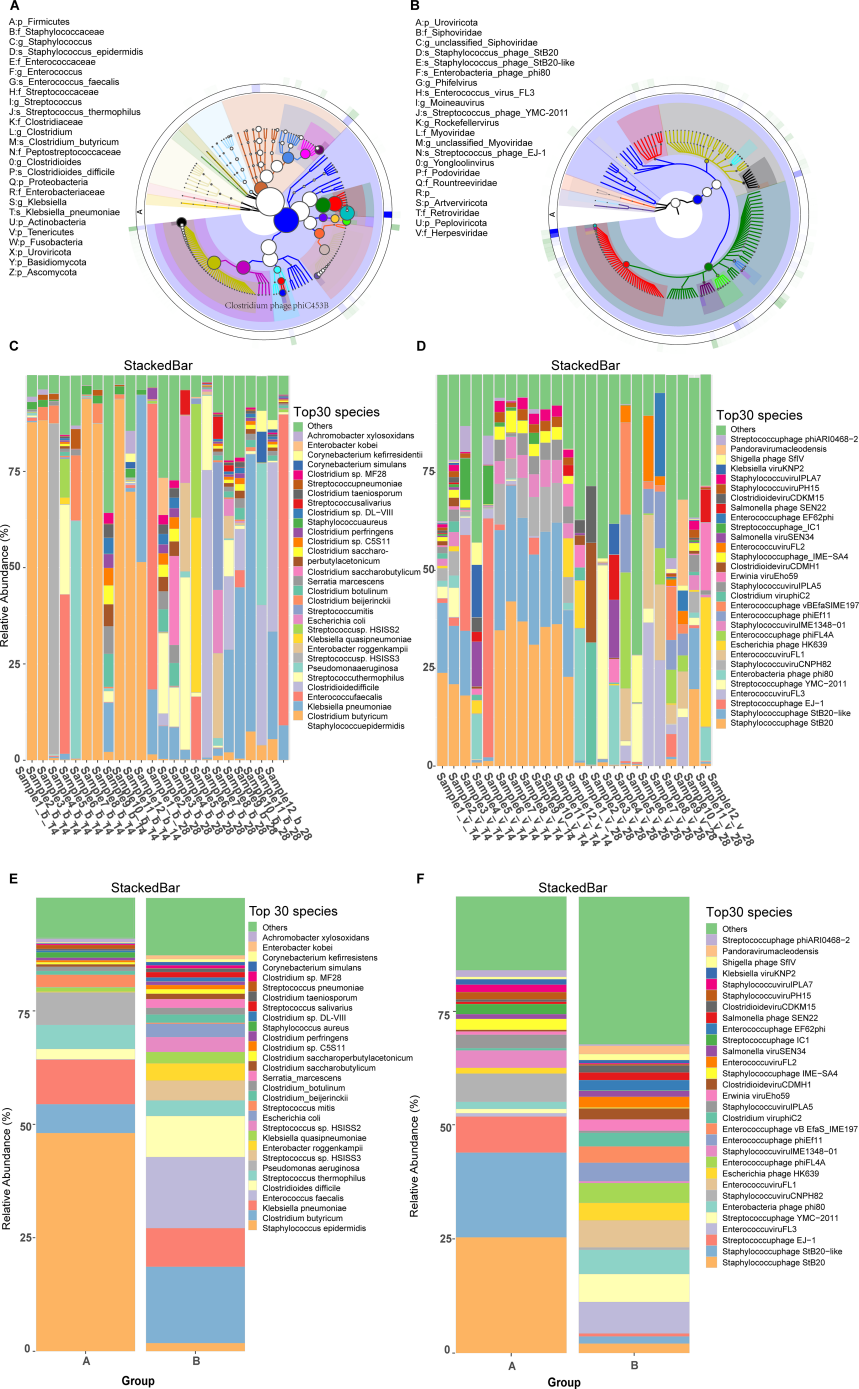
Microbial composition of preterm infant gut communities. **(A)** Composition of the species hierarchy of group bacterial communities. **(B)** Composition of the species hierarchy of group virus communities. Circular cladogram representing the phylogenetic tree of bacterial taxa. The different colors represent bacterial phyla, with each segment within the colored area indicating lower taxonomic levels such as genus and species. The size of the circles corresponds to the relative abundance of each taxon. Rings depict hierarchical taxonomic levels, moving from phylum (outer ring) to genus/species (inner rings). **(C)** Histogram of the composition of the species hierarchical structure of the sample bacterial community. **(D)** Histogram of the composition of the species hierarchical structure of the sample virus community. **(E)** Histogram of the composition of the species hierarchical structure of the group bacterial community. **(F)** Histogram of the composition of the species hierarchical structure of the group virus community.

To characterize the taxonomic composition of the intestinal microbiome in preterm infants, we annotated metagenomic reads using Kraken and Bracken (Lu et al., 2017). In addition, to thoroughly examine the impact of viruses on premature infants, we analyzed the composition of the viral populations in both groups using CAT (von Meijenfeldt et al., 2019) based on sequencing reads (**Figure 1B**). The top 150 viruses, ranked by community abundance, revealed that the annotated virome was overwhelmingly composed of viruses belonging to the phylum Uroviricota, which encompasses tailed bacteriophages of the order Caudovirales. Viral relative abundances reported in this study are expressed as proportions of the total quality-controlled sequencing reads per sample, ensuring that estimates reflect the true compositional context of the gut virome.

Viral relative abundances reported in this study are expressed as proportions of the total quality-controlled sequencing reads per sample, ensuring that estimates reflect the true compositional context of the gut virome. Due to limitations in current viral reference databases, a variable fraction of viral reads could not be taxonomically assigned across samples. While exact per-sample unclassified proportions were not retained in the final analysis tables, our intermediate processing logs indicate that annotation success ranged widely—approximately 40% to over 90% of viral reads were classified in different individuals. This implies that 10% to 60% of viral sequences remained uncharacterized, likely representing novel or underrepresented viral lineages in existing databases. This implies that 10% to 60% of viral sequences remained uncharacterized, likely representing novel or underrepresented viral lineages in existing databases. Despite this variability, all identified viruses belonged to the phylum *Uroviricota* (100% prevalence; median relative abundance: 0.14%), which encompasses tailed bacteriophages of the order *Caudovirales*. All taxonomically resolved viral sequences corresponded to bacteriophages infecting bacterial hosts such as *Staphylococcus*, *Streptococcus*, *Enterococcus*, and *Enterobacter*. Key species identified included *Staphylococcus phage StB20*, *Staphylococcus phage StB20-like, Streptococcus phage EJ-1, Enterococcus virus FL3*, and *Streptococcus phage YMC-2011*. The phylum-level prevalence and median relative abundances reported here were computed from the taxonomic profiles in **Supplementary Tables S5** (bacteria) and S6 (viruses). However, a portion of viral reads remained unidentified due to limitations in the reference databases, which primarily captured well-characterized viral species. This issue highlights the need for further expansion of viral reference databases, which will be addressed in future studies. These findings highlight the rich and varied microbial landscape within the intestinal microbiota of preterm infants, providing a foundation for future investigations into potential relationships between gut microbiota composition and clinical outcomes such as lung health.

The comparison of bacterial species composition between the 14- and 28-day groups revealed distinct distributions. The relative abundance of *Staphylococcus epidermidis* was significantly higher at 14 days (Paired t-test, *P* < 0.05), while *Enterococcus faecalis* was significantly higher at 28 days (Paired t-test, *P* < 0.05, **Figure 1C**; **Supplementary Table S5**). To provide a more transparent view of individual microbiome profiles, relative abundance profiles for each individual were displayed separately (**Figures 1C**, **D**). These individual profiles allow for a clearer understanding of the variations in microbial composition across different infants, showing that the abundance of species such as *Staphylococcus epidermidis* varied from negligible to over 50%, with some infants exhibiting more marked shifts in bacterial populations between the two time points. In terms of viral community relative abundance, there was a notable decrease in *Staphylococcus phages* from 14 to 28 days, whereas *Enterococcus phages* showed a general increase (**Figures 1E, F**; **Supplementary Table S6**). *Streptococcus phages*, which target and control *Streptococcus bacteria*, play a crucial role in maintaining a balanced gut microbiota by preventing bacterial overgrowth. These findings highlight the rich and varied microbial landscape within the intestinal microbiota of the study subjects, providing a robust foundation for further investigation into the role of specific microbial taxa in the health and disease states of preterm infants. This detailed microbial profiling is essential for understanding the intricate microbial ecosystems and their potential impacts on the clinical outcomes of this vulnerable population.

### Alterations in the diversity of communities of microbes

**Figure 2:**
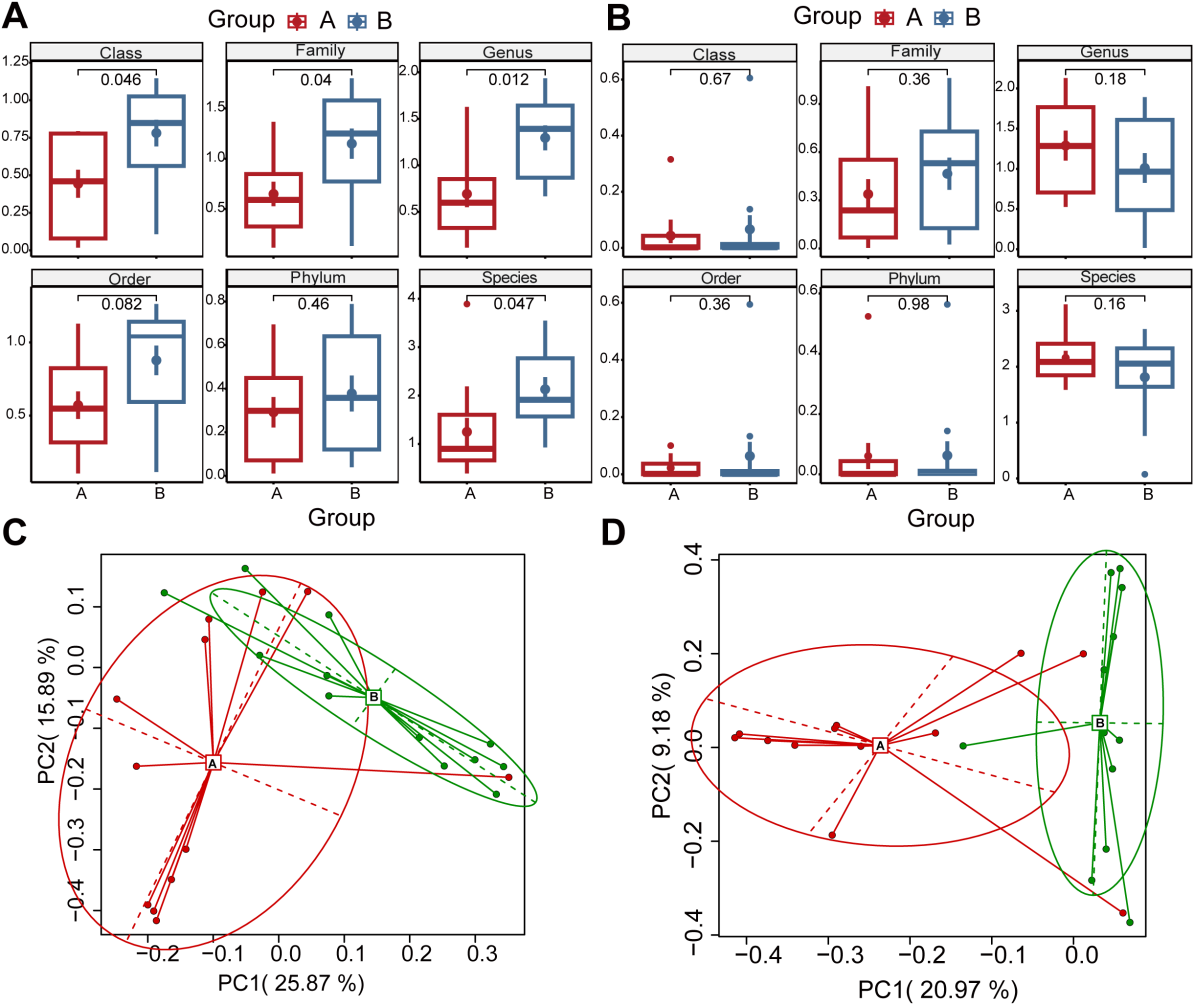
Alterations in the diversity of communities of microbes. **(A)** Bacterial structural Alpha diversity differences between groups; **(B)** Virus structural Alpha diversity differences between groups, The differences were assessed using the Shannon-paired T-test. **(C)** PCA analysis of bacterial community composition based on species; **(D)** PCA analysis of virus community composition based on species. For all the boxplots, the horizontal lines inside the boxes show the medians. Box bounds show the lower quartile (Q1, the 25th percentile) and the upper quartile (Q3, the 75th percentile). Whiskers are minima (Q1 − 1.5× IQR) and maxima (Q3 + 1.5× IQR), where IQR is the interquartile range (Q3–Q1).The error bars are given based on standard deviation of the mean (± SD).

Analysis revealed distinct differences in bacterial and viral community composition between 14- and 28-day postnatal time points. Alpha diversity, assessed using Shannon and Simpson indices, was significantly higher in bacterial communities at 28 days compared to 14 days (Shannon index: paired t-test, *P* < 0.05; **Figure 2A**; Simpson index: paired t-test, *P* < 0.05; **Supplementary Figure 1A**). Viral communities exhibited a non-significant increasing trend in alpha diversity at the family and species levels (Shannon index: paired t-test, *P* > 0.05; **Figure 2B**; Simpson index: paired t-test, *P* > 0.05; **Supplementary Figure 1B**). Beta diversity analyses based on Bray-Curtis dissimilarity and weighted UniFrac distances did not reveal statistically significant differences between the two time points for bacterial communities (**Supplementary Figure 2**) or viral communities (**Supplementary Figure 3**). Principal Component Analysis (PCA) on species-level relative abundances highlighted structural differences in both bacterial and viral communities between groups, as evidenced by distinct clustering patterns of samples (**Figures 2C, D**). Overall, microbial community composition underwent significant shifts from 14 to 28 days post-birth (**Supplementary Tables S6, S7**).

### Analysis of microbial composition relative abundance between groups

**Figure 3:**
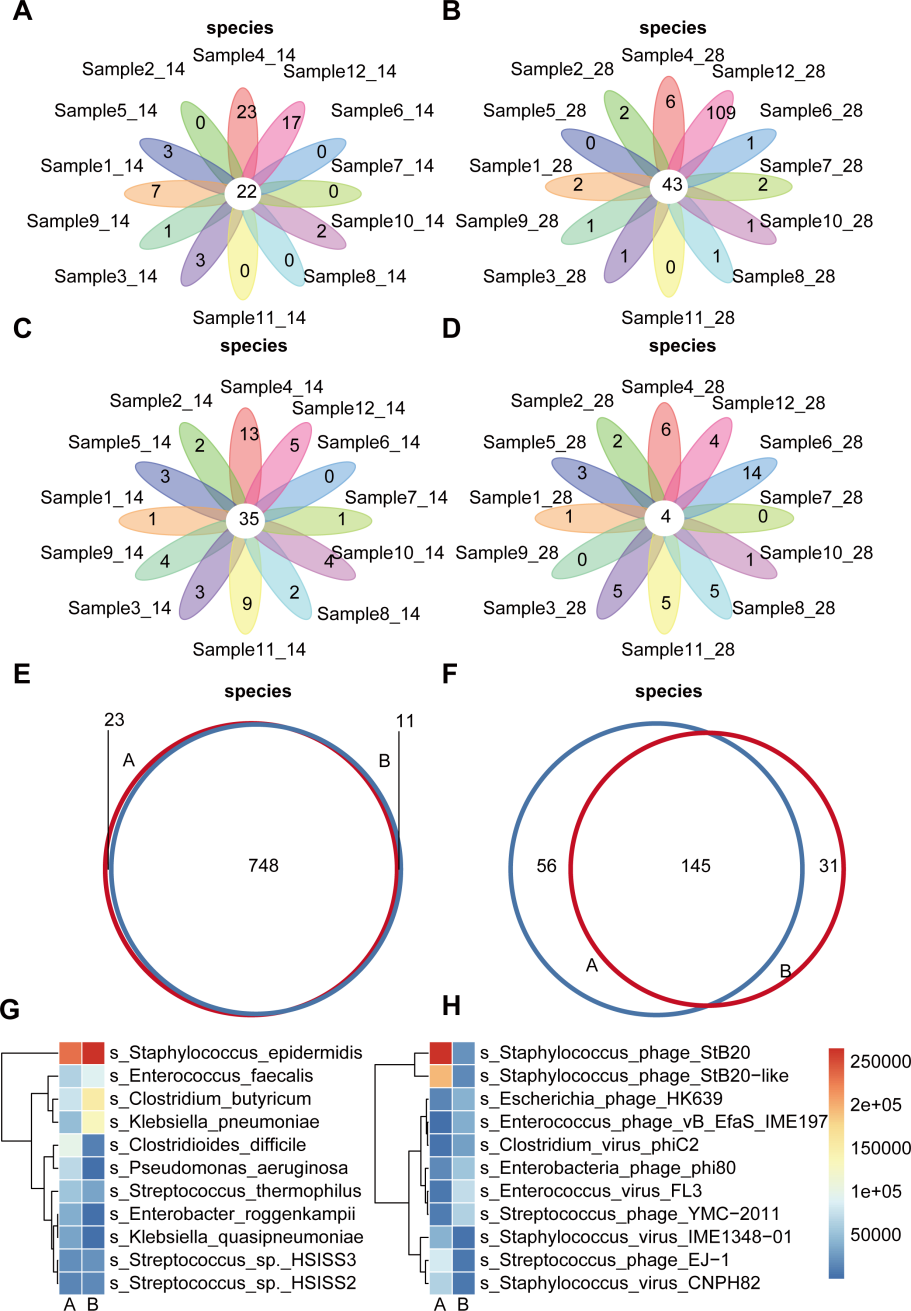
Analysis of microbial composition relative abundance between groups. **(A)** The flower plot of bacterial species of Group A **(B)** The flower plot of bacterial species of Group B **(C)** The flower plot of virus species of Group A **(D)** The flower plot of virus species of Group B The central circle represents the number of species shared by all samples, and the numbers on the petals represent the number of unique species in each sample. **(E)** The Venn graph of bacterial species between two groups. **(F)** The Venn graph of virus species between two groups. Each circle represents a group, and the numbers in the overlapping parts between circles represent the number of shared species between groups, the numbers outside the overlapping parts represent the number of unique species in each group. **(G)** Heatmap of clustering of the top 10 bacterial species in terms of relative abundance for species shared between groups. **(H)** Heatmap of clustering of the top 10 virus species in terms of relative abundance for species shared between groups.

To examine the distribution patterns and relative abundance of microbial species, we quantified shared and unique bacterial and viral species across inter-group and intra-group comparisons in this study (**Figure 3**). The bacterial species flower plot revealed that Group A contained 22 shared species, with approximately 41.67% of samples lacking unique species, 33.33% harboring fewer than 5 unique species, and only 25% exhibiting more than 5 unique species. In contrast, Group B exhibited 43 shared species, where 91.67% of samples contained limited unique species (**Figure 3A–B**; **Supplementary Table S8**). The analysis of viral species composition indicated that shared species outnumbered exclusive species in Group A, whereas Group B showed fewer shared species compared to unique species (**Figures 3C, D**; **Supplementary Table S9**). These findings suggest that the relative abundance of the bacterial community in the gut microbiota of preterm infants remains highly stable over time, consistent with previous observations (Maynard et al., 2012; Yatsunenko et al., 2012). The primary driver of bacterial diversity changes was identified as significant fluctuations in the relative abundance of bacterial taxa during development. Conversely, viral communities demonstrated marked variability both between and within groups, characterized by group/sample-specific viral species alongside shared members. For instance, 62.5% of viral species (145 out of 232 total) were shared between groups, while 37.5% (87 species) were group-specific. Intra-sample variations in shared and unique viral species were also evident (**Figures 3E–F**).

The 28-day gut-specific viral community included host-associated viruses such as Escherichia virus pro483, Megavirus chiliensis, and Spleen focus forming virus, which exhibit broad host specificity. These viruses regulate gut bacterial communities through lysis of host bacteria (e.g., *Clostridium*, *Enterococcus*, *Escherichia*). Additionally, bacteriophages like *Clostridium phage phiCT453A* and *Enterobacteria phage mEp460* may facilitate horizontal transfer of resistance or metabolic genes. Giant viruses, including *Megavirus chiliensis* and *Pandoravirus neocaledonia*, potentially interact with protists or larger microbial communities, influencing gut ecosystem stability. The presence of these viruses correlates with gut microbiota remodeling and host environmental adaptability.

Among bacteriophages, six species were consistently abundant across samples, while many low-abundance species highlighted the dynamic and individualized nature of the phage community.

The 14-day gut-specific viral community predominantly targets Gram-positive bacteria (e.g., *Propionibacterium*, *Streptococcus*), suggesting roles in early microbiota establishment and stabilization. Viruses like *Propionibacterium virus* series may aid in initial colonization via lysis-induced nutrient release. Similarly, *Pandoravirus dulcis* and *Pandoravirus salinus*, akin to 28-day giant viruses, likely regulate gut ecosystems. The 14-day viral community exhibits high diversity, including chimeric viruses (e.g., *Chimeric virus 14*) and Vibrio phages (e.g., *Vibrio phage ValB1MD 2*), indicating complex viral dynamics during early gut development. These viruses likely support microbiota establishment and host adaptation.

Shared viral species between 14- and 28-day samples include those infecting Gram-positive (Propionibacterium, Streptococcus) and Gram-negative bacteria (Escherichia, Salmonella). Their persistent presence suggests essential roles in maintaining core gut microbiome functions. These findings align with the hypothesis that viral communities dynamically reflect host-microbe interactions, environmental exposures, or antibiotic use (Hill et al., 2017; Lugli et al., 2023).

We ranked the relative abundance of shared and unique species between groups and selected top 10 species for clustering analysis (**Figure 3G–H**; **Supplementary Figures 6–7**; **Supplementary Tables S9–S10**). Microbial abundance differences between groups indicated relative stability in bacterial and viral communities at these time points, though denser sampling is required to confirm longitudinal trends. However, notable shifts in abundance structures likely contributed to diversity variations between groups. These results corroborate the view that early-life gut microbes gradually respond to internal/external influences, altering bacterial/viral community prevalence (de Muinck and Trosvik, 2018).

### Composition and function of the gut microbiota in preterm infants at 14 and 28 days

**Figure 4:**
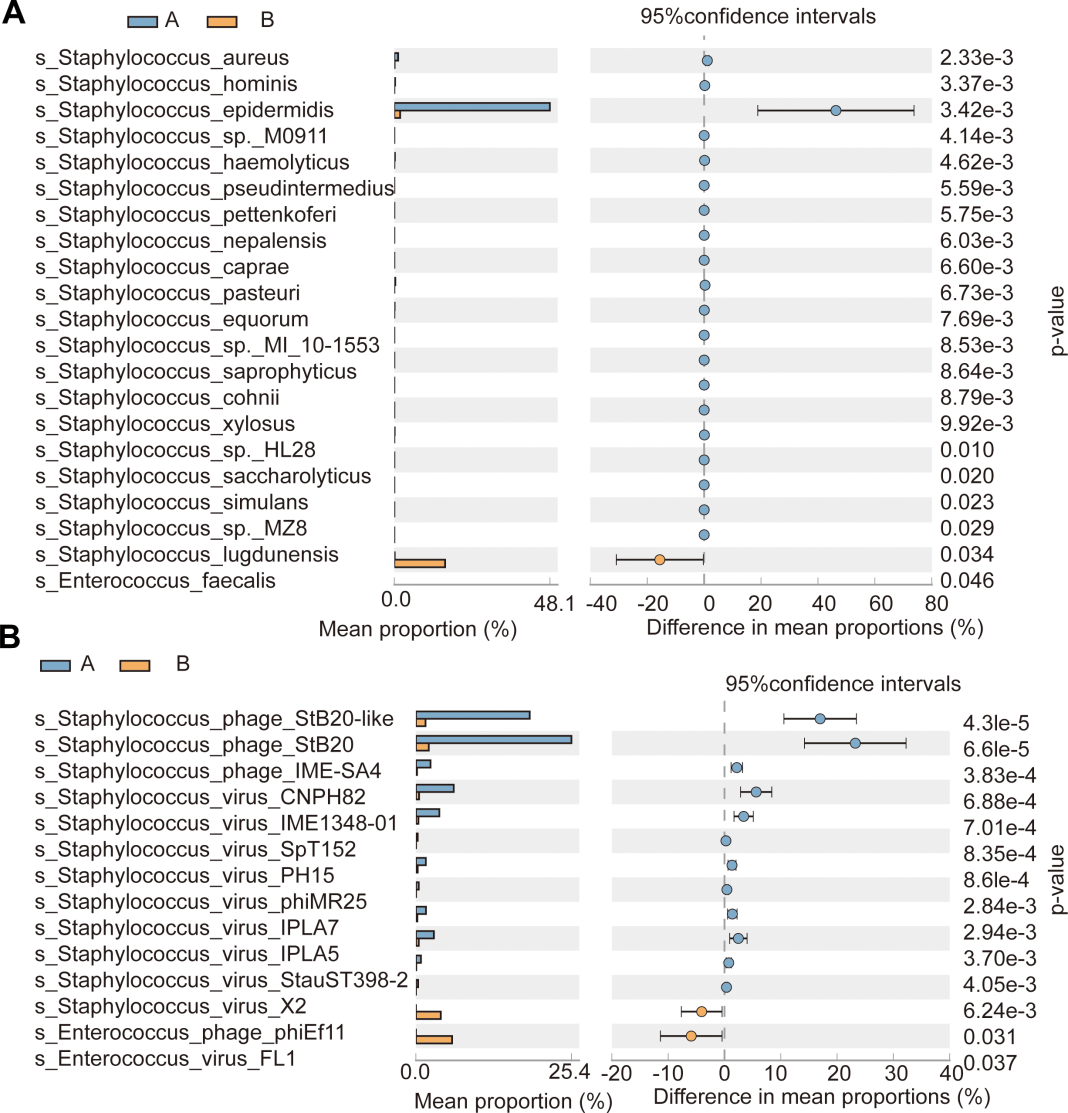
Analysis of composition of microbial communities: 14-day and 28-day premature infants **(A)** bacterial community composition; **(B)** virus community composition. The error bars on the columns represent standard errors (SE). All dots represent the percentage change in effect size between 14-day and 28-day preterm infants in bacterial community composition at 95% confidence intervals(CIs). Statistical significance was assessed using paired t-tests (*P* < 0.05). Mean values below 0 indicate a greater proportion in the bacterial community of 14-day preterm infants (blue dots; Group A), while mean values above 0 reflect a significantly greater proportion in the bacterial community of 28-day preterm infants(yellow dots; Group B).

The gut microbiota of preterm infants undergo dynamic changes during early development, consistent with previous studies documenting rapid microbial succession in neonatal gut ecosystems (Henderickx et al., 2019; Toubon et al., 2022; Westaway et al., 2022). To investigate these changes, we analyzed the relative abundance and diversity of bacterial and viral species in the gut microbiota of preterm infants at 14- and 28-day postnatal time points (**Figure 4**). Notable shifts were observed, including a reduction in Staphylococcus species prevalence, particularly a marked decline in *Staphylococcus epidermidis* relative abundance (**Figure 4A**; **Supplementary Table S12**), and a significant increase in *Enterococcus faecalis* abundance at 28 days compared to 14 days. These shifts may be attributed to factors such as antibiotic therapy (**Table 1**) and potential probiotic supplementation. For instance, *Lactobacillus rhamnosus-based probiotics* have been shown to enhance microbial diversity while reducing *Enterobacteriaceae* and *Staphylococcaceae* abundance, thereby promoting a healthier gut microbiota (Martí et al., 2021). The observed increase in *Enterococcus faecalis* may reflect its role as an opportunistic colonizer in the preterm gut. Given the underdeveloped microbiota and immune systems, its presence could represent a natural phase of microbial succession. However, further investigation into its functional impact on gut health and immune development is warranted.

Regarding viral communities, we observed changes in the relative abundance of *Staphylococcus-* and *Enterococcus-related bacteriophages* between 14 and 28 days (**Figure 4B**; **Supplementary Table S13**), reflecting fluctuations in their bacterial host populations. These findings underscore the dynamic phage-bacteria interactions within the gut microbiota. Although our analysis did not explicitly perform correlation tests between time and microbial shifts, paired t-tests confirmed statistically significant (*P* < 0.05) temporal changes in viral and bacterial abundance. These results highlight the age-dependent evolution of microbial and viral communities in preterm infants. We included a broad range of bacterial species, including low-abundance *Staphylococcus* sp*ecies* (excluding *S. epidermidis*) (**Figure 4**), to comprehensively capture microbial diversity and colonization dynamics. While these species may contribute minimally to overall microbiota composition, their inclusion provides a more complete picture of the early gut ecosystem, potentially revealing minor contributors critical to initial colonization. Additionally, even low-abundance species may play ecological roles or influence microbial balance, particularly in the context of immature immune systems in preterm infants. The proportion of unidentified viral reads in our dataset is detailed in **Supplementary Table S7**. Although most viral species were classified, a fraction of reads remained unassigned due to limitations in reference databases, emphasizing the need for expanded viral reference datasets in future studies to improve identification accuracy.

**Figure 5:**
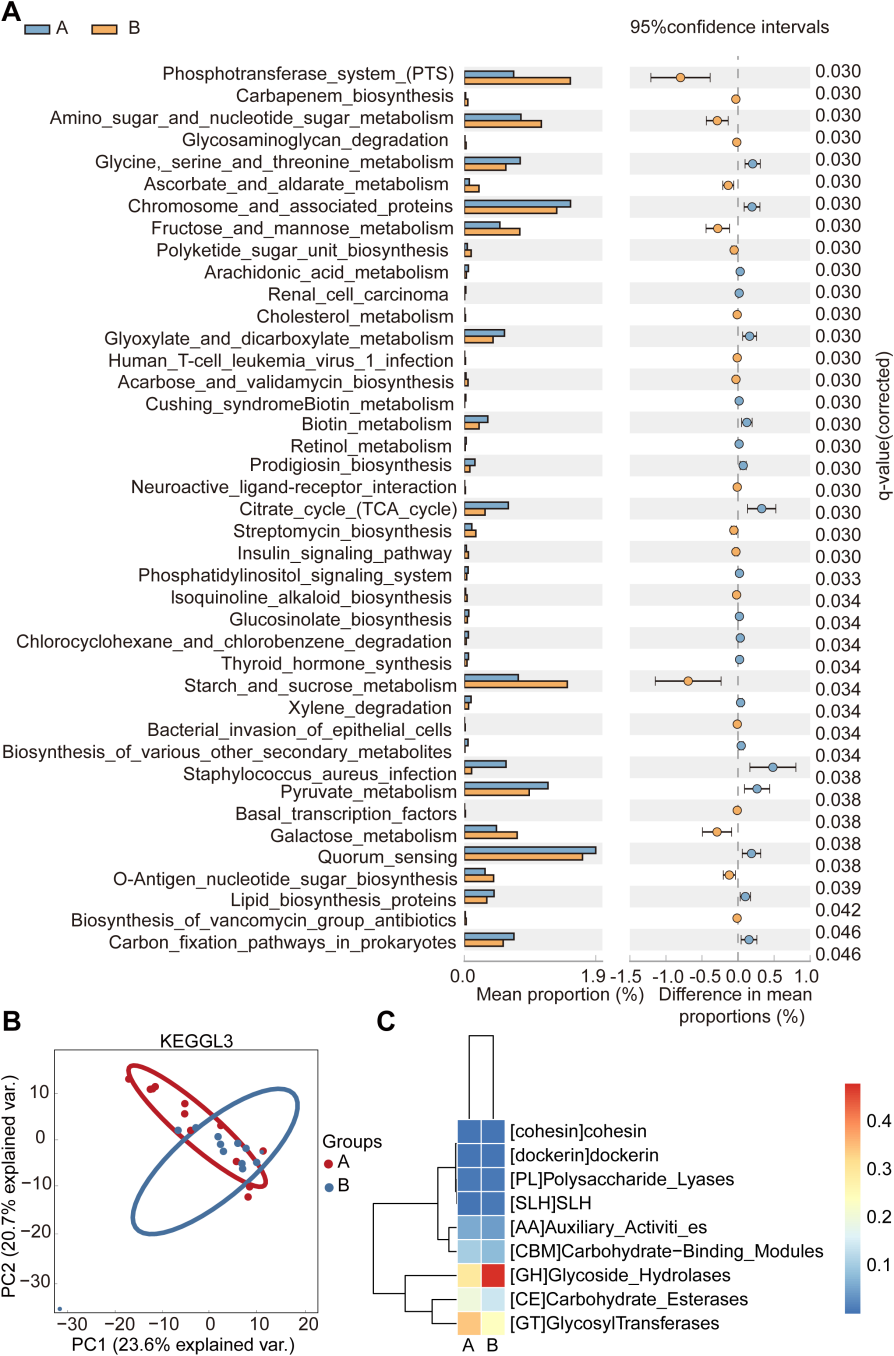
Analysis of functions of microbial communities: 14-day and 28-day premature infants. **(A)** bacterial community composition; **(B)** virus community composition. The error bars on the columns represent standard errors (SE). All dots represent the percentage change in effect size between 14-day and 28-day preterm infants in bacterial community composition at 95% confidence intervals (CIs). Statistical significance was assessed using paired t-tests (*P* < 0.05). Mean values below 0 indicate a greater proportion in the bacterial community of 14-day preterm infants (blue dots; Group A), while mean values above 0 reflect a significantly greater proportion in the bacterial community of 28-day preterm infants (yellow dots; Group B).

To further explore functional differences between bacterial and viral communities, we conducted KEGG pathway enrichment analysis (**Figure 5**). Among 21 significantly enriched pathways, those related to the phosphotransferase system (PTS) and starch/sucrose metabolism were prominent at 28 days, indicating adaptation to dietary carbohydrate utilization. In contrast, the Staphylococcus aureus infection pathway remained consistently low across both time points (**Figures 5A, B**; **Supplementary Tables S13–S14**). Statistical significance was determined using FDR (False Discovery Rate) correction, which controls false positives during multiple comparisons, ensuring reliable identification of significant pathways. Functional analysis of carbohydrate-active enzymes (CAZymes) revealed age-dependent shifts in microbial metabolic activity: Auxiliary Activities (AA), GlycosylTransferases (GT), Carbohydrate Esterases (CE), and Carbohydrate-binding Modules (CBM) predominated at 14 days, while Glycoside Hydrolases (GH) and Polysaccharide Lyases (PL) became more prevalent by 28 days (**Figure 5C**; **Supplementary Table S15**). These findings suggest developmental changes in carbohydrate metabolism, potentially influencing complex carbohydrate degradation and microbial ecology in the maturing gut (Loss et al., 2023).

### Analysis of functional differences in viral communities and phage characteristics

**Figure 6:**
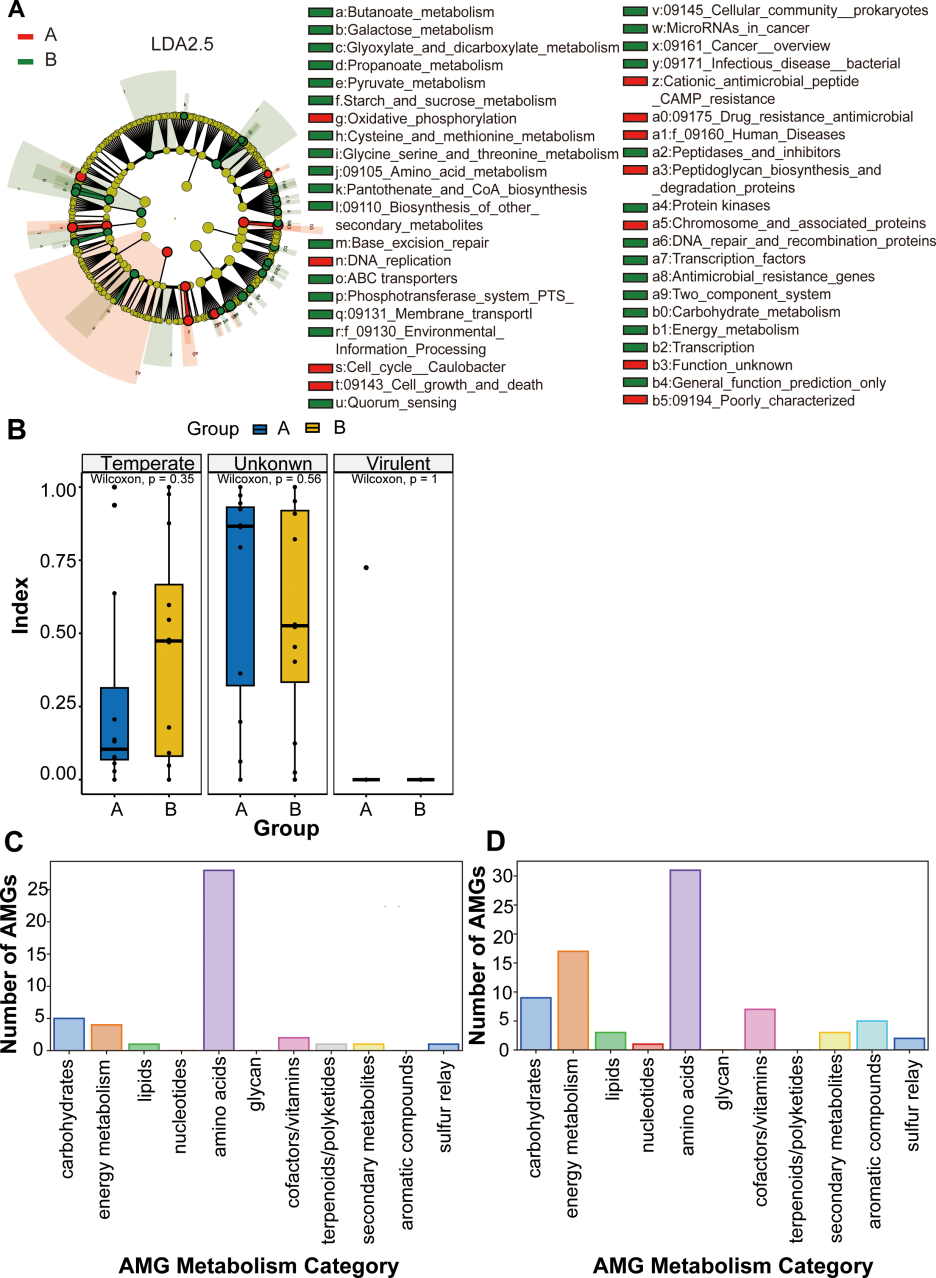
Analysis of functional differences in viral communities and phage characteristics. **(A)** Phylogenetic cladogram showing differentially abundant taxa from phylum to genus levels. Microbial classes are indicated with letters. Each node represents one taxon at different taxonomic levels; **(B)** Analysis of bacteriophage infectivity in Group A and Group B Error bars represent the standard deviation of the mean (± SD). Differences were assessed using the Wilcoxon paired test. **(C)** AMG metabolism categories for Group A; **(D)** AMG metabolism categories for Group **(B)** The vertical axis represents the number of AMGs included in each metabolic pathway, and the horizontal axis represents the corresponding metabolic entries.

To better characterize functional differences in viral community relative abundance between 14- and 28-day postnatal time points in preterm infants, we applied Linear Discriminant Analysis Effect Size (LEfSe) to identify significant functional biomarkers associated with microbial shifts. These biomarkers may offer insights into viral influences on gut health, though further research is needed to clarify their clinical relevance in preterm populations. In our study, LEfSe was used to analyze viral functional relative abundance differences in preterm infant gut microbiota across these two time points. The analysis began with non-parametric tests (e.g., Kruskal-Wallis rank-sum test) on viral functional data, followed by Linear Discriminant Analysis (LDA) to quantify effect sizes and compute LDA scores (**Figure 6A**; **Supplementary Table S16**). LEfSe results revealed that antimicrobial resistance and cationic antimicrobial peptide (CAMP) resistance pathways were significantly enriched at 14 days, while starch/sucrose metabolism, PTS, carbohydrate metabolism, and energy metabolism pathways dominated at 28 days. These findings correlated with bacterial community functional trends, suggesting auxiliary metabolism genes (AMGs) may mediate these shifts, consistent with bacterial functional relative abundance differences observed between groups. The dynamics of bacteriophage infectivity are critical for understanding microbial ecosystem stability. We examined phage infectivity patterns at 14- and 28-day postnatal periods and found a higher proportion of temperate phages at 28 days compared to 14 days. This suggests a temporal increase in temperate phage prevalence during this window. However, due to sampling limited to two time points, it remains unclear whether temperate phage proportions would continue to rise or decline beyond 28 days. Longitudinal studies with additional time points (e.g., 36 days or later) are necessary to establish long-term phage infectivity trends (**Figure 6B**). While low viral loads and limited sample sizes precluded statistically significant p-values, median value differences between groups were evident.

Bacteriophages, as viruses that replicate within bacterial hosts, often influence host metabolism through auxiliary metabolism genes (AMGs). These AMGs modulate bacterial metabolic pathways to support phage replication and propagation. We classified AMGs from both time points based on their involvement in metabolic processes (**Figures 6C, D**). Both groups showed dominant AMG activity in amino acid metabolism, including genes encoding enzymes for amino acid synthesis, degradation, or transport. At 28 days, carbohydrate, energy metabolism, and cofactor/vitamin pathways exhibited significant increases. While phage-related quorum sensing or cancer genes were not explicitly identified, our results suggest phages may alter host bacterial metabolism to facilitate replication and reshape the microbial ecosystem.

Notably, nucleotide metabolism and aromatic compound metabolism emerged in 28-day postnatal phage AMGs. Nucleotide metabolism genes (e.g., synthesis, degradation, or salvage pathways) and aromatic compound-metabolizing AMGs encode enzymes enabling phages to utilize, degrade, or modify aromatic substrates during host infection. In contrast, terpenoid/polyketide metabolism was prominent in 14-day postnatal phage AMGs. Although some AMGs encode terpenoid-degrading enzymes, terpenoids primarily function as secondary metabolites supporting microbial survival (e.g., antimicrobial defense, signaling) rather than directly fueling phage replication. The observed terpenoid decrease at 28 days likely reflects indirect effects on phage activity, such as ecosystem-level shifts or bacterial host availability, aligning with increased temperate phage prevalence at 28 days. Previous studies have shown that terpenoids regulate microbial interactions through antimicrobial and ecological effects (Dorman and Deans, 2000; Gershenzon and Dudareva, 2007; Zhou et al., 2024). Additionally, terpenoid-degrading AMGs may modulate host microbiota rather than directly provide phage energy (Hurwitz and U’Ren, 2016). More strikingly, most phage AMG categories showed increased abundance at 28 days compared to 14 days. These findings highlight dynamic changes in viral functional profiles, phage infectivity, and AMG activity in preterm infant gut microbiota during the first month of life. The observed shifts reflect adaptive responses to evolving microbial environments and metabolic demands of both viral and bacterial communities.

### Network analysis of bacteriophage-bacteria interactions and bacteria-clinical relevance

**Figure 7:**
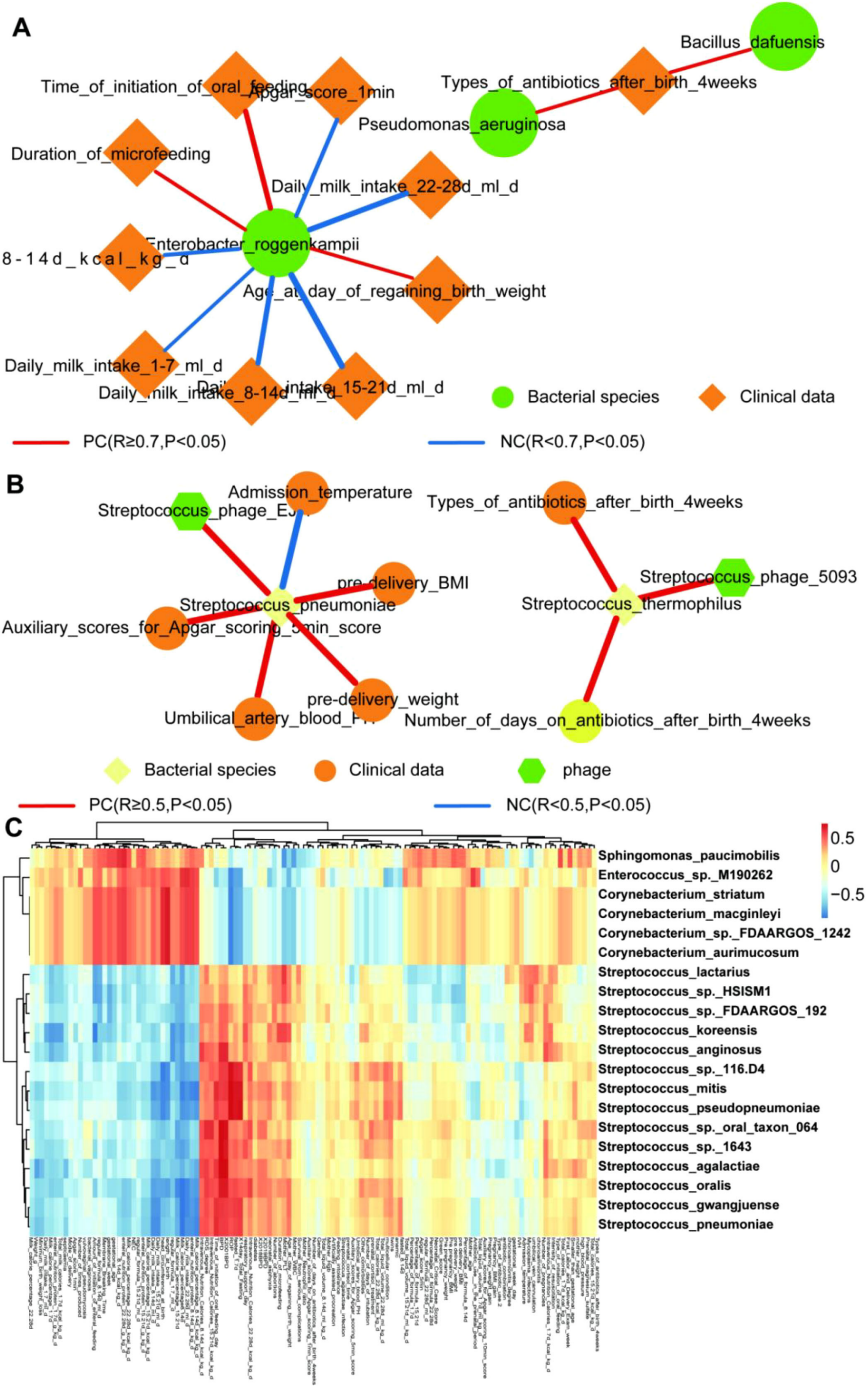
Network analysis of bacteriophage-bacteria interactions and bacteria-clinical relevance **(A)** Correlation network of bacterial species and clinical variables. Node colors represent bacterial species, while edge thickness indicates the strength of correlations. **(B)** Network showing interactions between bacteriophages, bacteria, and clinical variables based on Spearman correlation coefficients. Node sizes indicate the degree of connectivity, and edge colors represent the strength of correlations (red: positive correlations, blue: negative correlations). **(C)** Heatmap of the top 20 bacterial species ranked by the absolute sum of their correlation coefficients (absolute values) with all clinical data. Red indicates positive correlations, and Blue indicates negative correlations.

Predicting interactions between phages and clinical variables is crucial for advancing our understanding of microbial dynamics and their influence on health outcomes. Phages modulate microbial communities by specifically infecting and lysing bacterial hosts. To explore these interactions, we constructed a microbial interaction network linking clinical data from preterm infants with bacterial species, including 782 shared species across groups (**Figure 7A**; **Supplementary Table S17**). The *Enterobacter roggenkampii* network exhibited the strongest associations with daily milk intake (14–28 days post-birth) and oral feeding initiation timing. However, only 4 of the 12 infants had documented initiation times within this period, limiting the statistical robustness of this relationship. Notably, 7 out of 12 infants were twins, and microbiome data from twins may exhibit non-independence due to shared genetic and environmental factors, potentially introducing bias into statistical analyses. Given the small sample size and use of traditional statistical methods that neglect twin-related correlations, the impact of twin pairing remains a minor limitation. Future studies with larger cohorts, more comprehensive clinical datasets, and statistical frameworks accounting for non-independent twin data (e.g., mixed-effects models) are needed to validate these associations and clarify the role of oral feeding in shaping microbial dynamics.

As anticipated, antibiotic use within four weeks post-birth showed strong connections with *Pseudomonas aeruginosa*, a well-documented opportunistic pathogen linked to hospital-acquired infections and antibiotic resistance. Network analysis revealed significant associations between microbial species and clinical parameters: *Streptococcus thermophilus* demonstrated the strongest connectivity with post-four-week antibiotic duration and types, while *S. pneumoniae* exhibited weaker yet statistically significant correlations with admission temperature (**Figure 7B**). Furthermore, the heatmap of top 20 species highlighted key bacterial taxa associated with clinical variables, including milk intake and time to regain birth weight (**Figure 7C**). This analysis illustrates the complex interplay between microbial communities and clinical outcomes in preterm infants. However, *S. pneumoniae* was not among the top 20 species ranked by the absolute sum of correlation coefficients across all clinical variables (**Figure 7C**). These findings underscore the importance of elucidating phage-bacteria interactions and their clinical relevance to inform therapeutic strategies for preterm infants.

## Discussion

The interactions within the intestinal microbiota significantly influence host physiology, development, and immune function. Establishing a stable neonatal gut bacterial microbiome is critical, as it may shape the adult microbiome composition and affect long-term health outcomes, including obesity, inflammatory bowel disease, and food allergies (Cho and Blaser, 2012; Turnbaugh et al., 2009). Preterm infants face challenges such as gut microecological instability, delayed microbial colonization, and reduced microbial diversity, which increase disease susceptibility. However, postnatal succession patterns of gut microbiota (bacterial and viral) in preterm infants remain poorly characterized. Here, we comprehensively characterized the virome and bacterial communities in fecal samples from 12 preterm infants at 14- and 28-day postnatal time points to define postnatal microbial colonization dynamics. Our findings reveal key insights into dynamic microbial colonization processes and their potential implications for preterm infant health. Importantly, this study was designed as an exploratory analysis aimed at characterizing early temporal patterns in bacterial and viral community dynamics in preterm infants. Accordingly, the findings should be viewed as hypothesis-generating, providing a foundation for future targeted and mechanistic investigations rather than establishing definitive causal relationships. Future studies will be expand the sample size to include a higher proportion of non-twin participants. Moreover, the application of statistical approaches such as mixed-effects models will be essential to correct for potential genetic and environmental correlations within twin data and to strengthen the robustness of our findings. Despite these limitations, this exploratory study provides preliminary insights into bacterial–viral interactions during early gut development in preterm infants and highlights key microbial features that warrant validation in larger, hypothesis-driven longitudinal studies.

We observed significant inter- and intra-group variations in viral species composition between 14 and 28 days post-birth. These changes were most pronounced in the relative abundance of specific bacteriophages. For instance, *Staphylococcus phages* exhibited higher abundance at 14 days but significantly declined by 28 days, while *Enterococcus phages* increased during this period. This shift suggests that the infant gut virome dynamically responds to bacterial population changes, consistent with prior studies on phage persistence in preterm gut microbiota (Drall et al., 2019; Plummer et al., 2018). Furthermore, bacterial species such as *Enterococcus faecalis* and *Staphylococcus epidermidis* showed declining relative abundance, whereas *Clostridioides difficile* and *Klebsiella pneumoniae* remained stably present, supporting their role as core members of the early gut microbiota. Another limitation is the absence of a term-infant control group, which restricts our ability to distinguish preterm-specific microbial signatures from universal neonatal patterns. Future work will aim to incorporate term infant cohorts or conduct meta-analyses with published datasets to verify the specificity of observed taxa, such as the enrichment of *Enterococcus faecalis.*

The response of the virome to bacterial shifts highlights the intricate interplay between bacteria and phages. The observed decline in *Staphylococcus phages* and increase in *Enterococcus phages* may reflect phage-mediated regulation of bacterial populations, underscoring microbial homeostasis and the role of phage predation in shaping gut community structure in preterm infants. Additionally, the gut-lung axis is critical for understanding how gut dysbiosis influences systemic inflammation and lung development. The persistent presence of pathogens like *Clostridioides difficile* may contribute to inflammatory processes affecting lung health, a concern for preterm infants at high risk of respiratory complications. These findings emphasize the complex, individualized nature of the preterm virome, with distinct phage populations interacting with bacterial species in response to environmental and clinical factors (e.g., antibiotics, diet).

Notably, *Enterococcus faecalis* and *Staphylococcus epidermidis* showed significant declines in relative abundance between 14 and 28 days. 91.67% of samples exhibited reduced unique species richness, while Sample12 contained 109 unique bacterial species at 28 days. We performed quality control checks (adapter trimming, low-quality read removal, contamination screening) to exclude technical artifacts (see Methods). A plausible explanation for this anomaly is transient bacterial colonization driven by external factors such as diet, medical interventions, or environmental exposure. However, additional biological replicates and detailed metadata would be necessary to confirm the underlying cause of this outlier.

The relative abundance of *Clostridioides difficile* and *Klebsiella pneumoniae* remained stable between 14- and 28-day time points, suggesting their potential role as core members of the early gut microbiota. Nonetheless, the dynamic nature of the preterm gut microbiome, characterized by ongoing maturation and environmental influences, necessitates further investigation with additional time points to confirm the long-term stability of these species. The observed patterns of significant inter-sample variation in phage abundance, limited shared species, and high prevalence of unique phage taxa align with established virome research, particularly in gut microbiota studies. Phage communities are highly individualized, strongly shaped by host microbiome composition. As noted by Norman et al. (2015), inter-individual variability in phage populations is substantial, driven by host-specific microbial ecosystems. Most phages exhibit host specificity, inherently limiting cross-individual overlap in shared species. Our findings corroborate prior evidence that *Staphylococcus phages* regulate bacterial populations through lysis of *Staphylococcus* species, thereby preventing overgrowth and maintaining microbial homeostasis (Guerin et al., 2018; Minot et al., 2011). The presence of numerous unique phages in our samples may reflect transient interactions or conditions (e.g., dietary shifts, immune responses, or bacterial community dynamics), consistent with studies documenting dynamic phage-bacterial interactions in the gut (Shkoporov et al., 2019). The high proportion of unique phage species may also stem from technical limitations in current virome databases, as many phages remain uncharacterized. Advancements in reference databases and annotation tools could reduce observed uniqueness. Gut environments exhibit substantial inter-individual variation, influenced by diet, antibiotic exposure, mode of delivery, and immune status, all of which contribute to distinct phage compositions. Our results highlight the highly dynamic, personalized, and diverse nature of gut phage populations, consistent with current virome research paradigms. Nevertheless, clinical metadata such as detailed antibiotic types, duration of administration, and comprehensive feeding practices were incompletely recorded in this study. To strengthen causal inference, future research should systematically collect these data and apply multivariate regression frameworks to control for confounding factors including birth weight, feeding mode, and antibiotic exposure.

The potential involvement of Ascomycetes in propionic acid fermentation via the propylene glycol pathway in early-life microbiota has been reported (Cerdó et al., 2018). Prenatal and postnatal antibiotic exposure alters microbial colonization patterns, potentially affecting microbiota development (Forsgren et al., 2017; Gibson et al., 2016). During the transition from breastfeeding to weaning, the newborn gut microbiota undergoes shifts in dominant taxa, with *Firmicutes* and *Bacteroides* being replaced by *Proteobacteria* and *Actinobacteria* (Koenig et al., 2011). Preterm infants lacking breastfeeding and receiving intensive antibiotic therapy between 14 and 28 days post-birth exhibit a marked increase in *Proteobacteria*. Analysis of bacterial and viral community structures corroborates prior observations (Hesla et al., 2014). This finding aligns with a study describing fecal microbiome composition in preterm infants under 42 days of age, which demonstrated that preterm gut microbiota exhibit dynamic changes across time points (Khan et al., 2023). Despite fluctuations in relative abundance, certain core taxa remained consistently present, indicating compositional stability at the species level. Among KEGG pathways analyzed in the gut microbiota between 14 and 28 days post-birth, those related to carbohydrate metabolism, amino acid metabolism, and energy metabolism showed significant differences between time points, reflecting dynamic shifts in microbial activity as the gut microbiota matures. In contrast, infection-related pathways, such as the *Staphylococcus aureus* infection pathway, maintained consistently low abundance without significant variation, suggesting minimal contribution during this phase of microbial colonization in preterm infants (**Figure 5**).

The immature intestinal and skin barriers of preterm infants, together with their underdeveloped immune systems, may increase susceptibility to viral infections. Viral infections could predispose them to respiratory illnesses, gastrointestinal disorders, and other complications, increasing medical risks (Morrow et al., 2013; Normann et al., 2013). Our study revealed significant differences in viral community functional profiles and bacteriophage characteristics between 14- and 28-day-old preterm infants (**Figure 6**). These findings suggest that preterm gut microbiota may experience dysbiosis, further elevating infection risks.

A recent study demonstrated that viral colonization in early life follows a gradual progression (Liang et al., 2020). The initial phase involves phage activation by pioneer bacteria, followed by viral colonization of infected human cells. The timing of breastfeeding initiation is the primary determinant of this process, with preterm infants typically initiating later than term newborns. Additionally, individual correlations and age significantly influence viral diversity, while environmental exposure remains a critical factor affecting viral abundance (Lim et al., 2015). We observed increased viral richness in the gut at 28 days compared to 14 days post-birth (**Figure 3**). However, no significant rise in unique viral species per sample was detected, and we hypothesize this may reflect interindividual variability, consistent with studies using *enteroviruses* to identify pre-symptomatic signatures of *necrotizing enterocolitis* in preterm infants. Notably, high inter-individual variability exists among enteroviral groups in preterm infants, whereas intra-individual variability over time remains relatively low (Kaelin et al., 2022). A longitudinal study tracking gut bacterial development in 12 infants during their first year revealed that microbial communities interact continuously with internal and external factors from uterine colonization onward. These interactions drive fluctuations in phylum-level relative abundance, such as increased *Firmicutes* and *Proteobacteria*, resulting in nonlinear microbial succession (de Muinck and Trosvik, 2018). Viruses are known to manipulate host cellular processes to enhance replication and propagation. Beyond core genes for replication and assembly, some viruses encode auxiliary metabolic genes (AMGs) that modulate host metabolism to create favorable environments for proliferation. We hypothesize that AMGs related to carbohydrate metabolism and the phosphotransferase system (PTS) may enhance nutrient utilization in bacterial hosts such as *E. faecalis*, providing them with colonization advantages. These functional shifts may also influence host nutrient absorption and immune development, although further experimental validation will be required to substantiate these mechanistic links. Future research should focus on functionally validating terpenoid-degrading enzymes encoded by AMGs and their ecological impacts to elucidate mechanisms underlying these interactions, potentially informing novel antiviral strategies. By analyzing metabolic profiles of phages and bacteria with a focus on amino acid metabolism (**Figure 7**), we identified key pathways and interactions shaping phage-bacterial relationships. Our network analysis highlights amino acid metabolism as a critical driver of phage-bacterial interactions, offering clinical insights into these complex microbial dynamics.

Bacteriophages, viruses that specifically infect bacteria, may also play a significant role in the intestines of premature infants. Studies suggest that bacteriophage communities in the intestines of preterm infants differ from those of full-term infants, potentially due to factors such as immature immune systems and antibiotic exposure (McElroy et al., 2013).Understanding the roles of viruses and bacteriophages in preterm infant intestines and their associations with health outcomes is critical for optimizing clinical care. Our results demonstrate dynamic temporal shifts in bacterial and viral relative abundances, including a decline in *Staphylococcus-associated* phages and an increase in *Enterococcus-associated* phages between days 14 and 28, as microbial composition evolves. These trends suggest phage communities may respond to bacterial population changes, though functional studies are needed to clarify adaptive mechanisms. For instance, the decline in *E. faecalis* and *S. epidermidis* alongside increases in their respective bacteriophages implies phage predation may regulate bacterial dynamics. Such interactions are essential for understanding how gut microbiota maintains homeostasis and adapts to environmental perturbations, particularly in the vulnerable preterm gut ecosystem. Alongside compositional shifts, significant functional reorganization occurred in the gut microbiota. The decrease in *E. faecalis* and *S. epidermidis* and rise in *Enterococcus bacteriophages* may indicate altered roles in nutrient metabolism, immune regulation, and barrier function. Notably, the increase in *Clostridium butyricum*, a *butyrate-producing* species, suggests a transition toward a more beneficial metabolic profile supporting gut health and immune maturation. These dynamic bacterial and viral changes underscore the importance of balanced gut microbiota for preterm infant well-being. The limited sampling in the current study, restricted to two time points (14 and 28 days), hinders the ability to determine whether the observed increase in temperate phages at 28 days represents a sustained trend or a transient fluctuation Future investigations incorporating additional time points (e.g., 36 days or later) could provide more comprehensive insights into temperate phage dynamics in preterm gut microbiomes. Additionally, this study focused exclusively on preterm infants without including full-term controls, limiting comparisons between preterm and term microbial dynamics. While our findings highlight temporal changes within the preterm cohort, future studies with full-term control groups are necessary to better characterize preterm gut microbiota uniqueness. Furthermore, the relatively small cohort size (n=12) presents a major limitation, especially considering numerous confounding factors listed in **Table 1** (gestational age, birth weight, feeding practices, antibiotic exposure). To better capture the early colonization events and longer-term microbial trajectories, future studies should employ denser longitudinal sampling schedules, including time points within the first week of life and beyond 28 days.

These variables may influence observed microbial patterns, and the small sample size restricts robust statistical analysis. Thus, caution is warranted when generalizing these findings. Future studies with larger cohorts and expanded metadata collection are essential to validate these preliminary observations and disentangle confounding effects on gut microbiota development in preterm infants. Disruptions in early microbial communities could increase infection susceptibility, inflammatory risks, and long-term health consequences. Understanding these dynamics may inform clinical strategies to promote gut health, such as targeted probiotics or bacteriophage therapies. Although this study analyzed preterm gut microbiota, it did not specifically examine associations between microbial taxa and lung injury outcomes like *bronchopulmonary dysplasia* (BPD). Future research with larger cohorts and targeted analyses will be needed to elucidate these relationships in greater depth.

It is well-established that diet, antibiotic exposure, and delivery mode significantly shape the bacterial microbiome composition (Bäckhed et al., 2015; Cho and Blaser, 2012; Dominguez-Bello et al., 2010; Koenig et al., 2011; La Rosa et al., 2014; Turnbaugh et al., 2009). Although our cohort included preterm infants with variations in labor duration, treatment protocols, and immune status, the small sample size limited the ability to evaluate the impact of these factors on microbial composition. We included DNA viruses in this study due to the use of a DNA-centric extraction and sequencing methodology. RNA viruses, which lack DNA, were not captured in our analysis, resulting in the exclusion of a significant portion of the virome, including *coronaviruses*, *picornaviruses*, and *rotaviruses*. RNA viruses play pivotal roles in shaping gut microbiome dynamics, particularly during neonatal development, and their omission limits the comprehensiveness of virome characterization.

To address this limitation, future studies should employ RNA sequencing approaches, such as metatranscriptomics, to detect RNA viruses and achieve a more holistic virome profile. A dual extraction protocol capturing both DNA and RNA would further enable simultaneous analysis of DNA viruses, RNA viruses, and active microbial transcription, offering a more complete understanding of microbial-viral interactions in the infant gut. Furthermore, while this study compares microbial community composition at two time points (14 and 28 days), it does not constitute a fully longitudinal investigation. The neonatal gut microbiome is highly dynamic, and robust conclusions about temporal trends require denser sampling across multiple time points. Although our findings suggest relative stability between these two time points, studies with higher temporal resolution are needed to validate this observation.

Despite these limitations, this study provides valuable insights into bacterial-viral interactions during early gut development. We emphasize the critical role of these microbial dynamics in preterm infant health and development, underscoring the need for longitudinal studies with larger cohorts to confirm our findings and clarify underlying mechanisms. Oral probiotic supplementation represents a promising strategy to promote beneficial gut colonization (Onyango et al., 2021). In preterm infants born via cesarean section, daily administration of *Lactobacillus rhamnosus* has been shown to diversify the gut microbiota and reduce *Enterobacteriaceae* and *Staphylococcaceae* abundance (Martí et al., 2021). While our study did not directly examine probiotic effects, the observed shifts in microbial diversity and abundance align with outcomes from probiotic interventions. These findings highlight the potential of targeted probiotic therapies to foster healthy gut microbiota development in preterm infants. Additionally, exploring therapeutic approaches such as precision probiotics or phage-based interventions may offer innovative strategies to support gut microbiome maturation in this vulnerable population.

## Methods

### Study design and sample collection

The collected fecal samples were immediately sent to the laboratory on the same floor. The stool was divided into 2–4 tubes based on the amount collected, placed in sterilized Eppendorf tubes, and then stored in the laboratory –80°C refrigerator for further Uniform DNA extraction of fecal microbiota. This study was approved by the Shenzhen People’s Hospital Institutional Review Board (LL-KY 2022288).

### Metagenome and metavirus library construction

For metagenome library construction, 1 µg of extracted genomic DNA was fragmented to an average size of ~250 bp using a Bioruptor sonicator (Diagenode). The fragmented DNA was then subjected to end repair, blunting, and phosphorylation using T4 DNA polymerase, Klenow Fragment, and T4 polynucleotide kinase. The DNA fragments were then 3’ adenylated using the Klenow Fragment (3’-5’ exo-) and subsequently ligated to adaptors using T4 DNA Ligase. After each reaction step, the DNA was purified using the QIAquick PCR purification kit (Qiagen).The final libraries were generated by PCR amplification using adapter-compatible barcode primers. For Metavirus, samples were resuspended in 10ml of SM buffer and homogenized. After 5min chilling on ice, the samples were centrifuged at 5000rpm for 10min at 4°C. The supernatants were transferred to a new tube and centrifuged again. The supernatants were then filtered twice with a 0.45um filters. A final concentration of 0.5M NaCl and 10% of PEG8000 were then added, and incubated overnight at 4°C. Then the samples were centrifuged at 5000rpm for 20min at 4°C, collecting the precipitate. Pellets were resuspended in 400ul of SM buffer and the same volume of chloroform. After emulsification, the samples were centrifuged at 2500g for 5min. The aqueous phase was transferred into a new tube and mixed with DNase and RNase incubating at 37°C for 1hour. VLP nucleic acid was then extracted using QIAamp Viral RNA Mini Kit according to the manufacture’ s instructions. After reverse transcription for RNA nucleic acid, both DNA and cDNA were amplified using MDA technology, respectively. The amplified dsDNA was then used for library construction by E-GENE Tech Co., Ltd. Both libraries were quantified with an Agilent 2100 Bioanalyzer (Agilent Technologies) and real-time PCR assays. Sequencing was performed at E-GENE in Shenzhen using the Illumina sequencing platform.

### Metagenome assembly and taxonomy annotation

Based on unique barcode and primer sequences, paired-end reads were accurately mapped to their respective samples. High-quality clean reads were generated by removing adapter sequences and low-quality reads using Trimmomatic (Bolger et al., 2014, v0.38)(V0.38). with the following parameters: SLIDINGWINDOW:5:15, HEADCROP:3, AVGQUAL:15, LEADING:5, TRAILING:5, MINLEN:80. Sequencing was performed on the Illumina NovaSeq 6000 platform using 150-bp paired-end reads, employing Illumina’s latest S4 flow cell chemistry and reagent kits to ensure high throughput and data accuracy. To monitor contamination, kit-negative controls (no-template controls) and sequencing-negative controls (blank library preparations) were processed in parallel with experimental samples during DNA extraction, library construction, and sequencing. Analysis of control data revealed negligible read counts, confirming minimal contamination risk. For metagenomic sequencing data, clean reads were assembled into contigs using MEGAHIT (v1.0.6) (Li et al., 2016).Host DNA was removed via the NCBI Human Sequence Removal protocol using a human reference genome (hg38). Taxonomic annotation was performed using Kraken2 & Bracken with the Kraken2 Virus database. Metavirome data were processed using identical methods: host DNA was filtered using the NCBI Human Sequence Removal protocol with hg38 reference, reads were assembled into contigs via MEGAHIT (v1.0.6), and taxonomic classification was conducted using CAT (v5.3.2) through the LCA (lowest common ancestor) algorithm.

### Metavirome diversity analysis

Alpha and beta diversity analyses were conducted to assess microbial shifts between 14 and 28 days post-birth. Alpha diversity, reflecting species richness and evenness within each sample, was calculated using the Shannon index. Statistical comparisons of alpha diversity between the two time points were performed using paired t-tests. Beta diversity was analyzed using two complementary approaches: Bray-Curtis distance, which measures compositional dissimilarity based on relative abundance, and weighted UniFrac distance, which accounts for phylogenetic relationships among taxa. Differences in beta diversity were assessed using ANOSIM (Bray-Curtis distance) and PERMANOVA (Permutational Multivariate Analysis of Variance). ANOSIM provided an R-value (ranging from −1 to +1),where 0 suggests the null hypothesis cannot be rejected (Clarke, 1993)), where 0 suggests no difference between groups, and PERMANOVA provided an R² value (ranging from 0 to 1), quantifying the proportion of variation explained by group differences. Sequencing depth normalization was conducted using rarefaction, and data were transformed using centered log-ratio (CLR) transformation for beta diversity analysis. Differences in community structure based on beta diversity were visualized using principal coordinate analysis (PCoA) with the ape package and non-metric multidimensional scaling (NMDS) with the VEGAN package(v2.5-3) (Oksanen et al., 2015), both using Bray-Curtis distance. Statistical significance for both PCoA and NMDS was assessed using PERMANOVA. Phylum, genus, and species differences between groups were identified using STAMP (v2.1.3) (Parks et al., 2014), with Welch’s t-test used for statistical comparisons. All analyses defined statistical significance as *P* < 0.05.

### Host prediction of phage

1. Based on Spacer sequence library in CRISPRCasdb database, Blastn-short alignment of assembled contig sequences was performed to screen phage candidate host information. The screening threshold is e-value ≤ 1e-10 and the identity is ≥ 95%.
2. Based on the phage and microorganism relationship database Microbe Versus Phage (MVP, http://mvp.https://ngdc.cncb.ac.cn/databasecommons/), the similarity between the assembled contig and the virus sequences in the database was determined by alignment, and the possible hosts corresponding to the contig were estimated by relational database mapping results.

### Metavirome nonredundant gene set prediction and annotation

Open reading frames (ORFs) were predicted by prodigal (v2.6.3) form contigs and the clean reads were aligned to ORFs to calculate the mapped read count. Thereafter, the ORFs were clustered to remove redundancy for building a set of representative genes through CD-HIT software (Fu et al., 2012) (v4.7). To avoid bias related to variation in the ORF size, both ORF sequence length and sequencing depth were included in the data normalization process prior to statistical comparisons (Hu et al., 2020). The normalized abundance matrix (G) was calculated for all samples:


$$G={\left(g_{kh}\right)}_{m\times n}=\left[\begin{array}{ccc} g_{11} & \ldots & g_{1m}\\ \vdots & \ddots & \vdots \\ g_{n1} & \ldots & g_{nm} \end{array}\right],\;g_{kh}=\frac{\sum_{i=1}^{p}\frac{D_{ki}}{L_{ki}}}{\sum_{h=1}^{n}\sum_{i=1}^{p}\frac{D_{ki}}{L_{ki}}}$$

where m is the number of samples, n is the number of representative genes, gkh is the normalized abundance of representative gene h for sample k, p is the total number of ORFs clustered for representative gene h and sample k, Dki is the mapped reads count of ORF i for sample k, and Lki is the length of ORF i for sample k.

ORFs were annotated with functional information using the Annotation softwarer KofamKOALA of Kyoto Encyclopedia of Genes and Genomes (KEGG) (Aramaki et al., 2020) based on the KEGG database with default parameters. COG annotation of ORFs was performed using eggnog-emapper software. ARGs were characterized by mapping ORFs to the Comprehensive Antibiotic Resistance Database (CARD) (V3.0.0) using the Resistance Gene Identifier (RGI) application. The CARD is a rigorously curated collection of characterized, peer-reviewed antibiotic resistance determinants (McArthur et al., 2013). Diamond was used to map the ORFs to the MGE database. The MGE database contains genes with 278 different gene name annotations (annotated as IS*, ISCR*, intI1, int2, istA*, istB*, qacEdelta, tniA*, tniB*, tnpA*, and Tn916 transposon in the NCBI nucleotide database) and more than 2000 unique sequences, excluding the sequences from the PlasmidFinder database (Pärnänen et al., 2018). Subsets of ORFs that were mapped to MGEs were used to produce the MGE profiles. The analysis of Metagenome also refer to this method.

### Statistical and network analysis

Spearman correlation coefficients were calculated to assess the relationships between microbial species and clinical variables. Correlation networks were constructed focusing on interactions with |R| > 0.7 and *P* < 0.05.

### Correlation analysis

The correlation analysis between virus and clinical data was performed using R package corrplot.

## Data Availability

The datasets presented in this study can be found in online repositories (Chen et al., 2021). The names of the repository/repositories and accession number(s) can be found in the article/**Supplementary Material**.
