# Supplementary figures and images for: Temporal dynamics of gut microbiota and virome in preterm infants: insights from longitudinal metagenomic analysis
Source: Front Cell Infect Microbiol. 2026 Mar 9;16:1598786. doi: 10.3389/fcimb.2026.1598786 (PMC13006629; doi:10.3389/fcimb.2026.1598786)

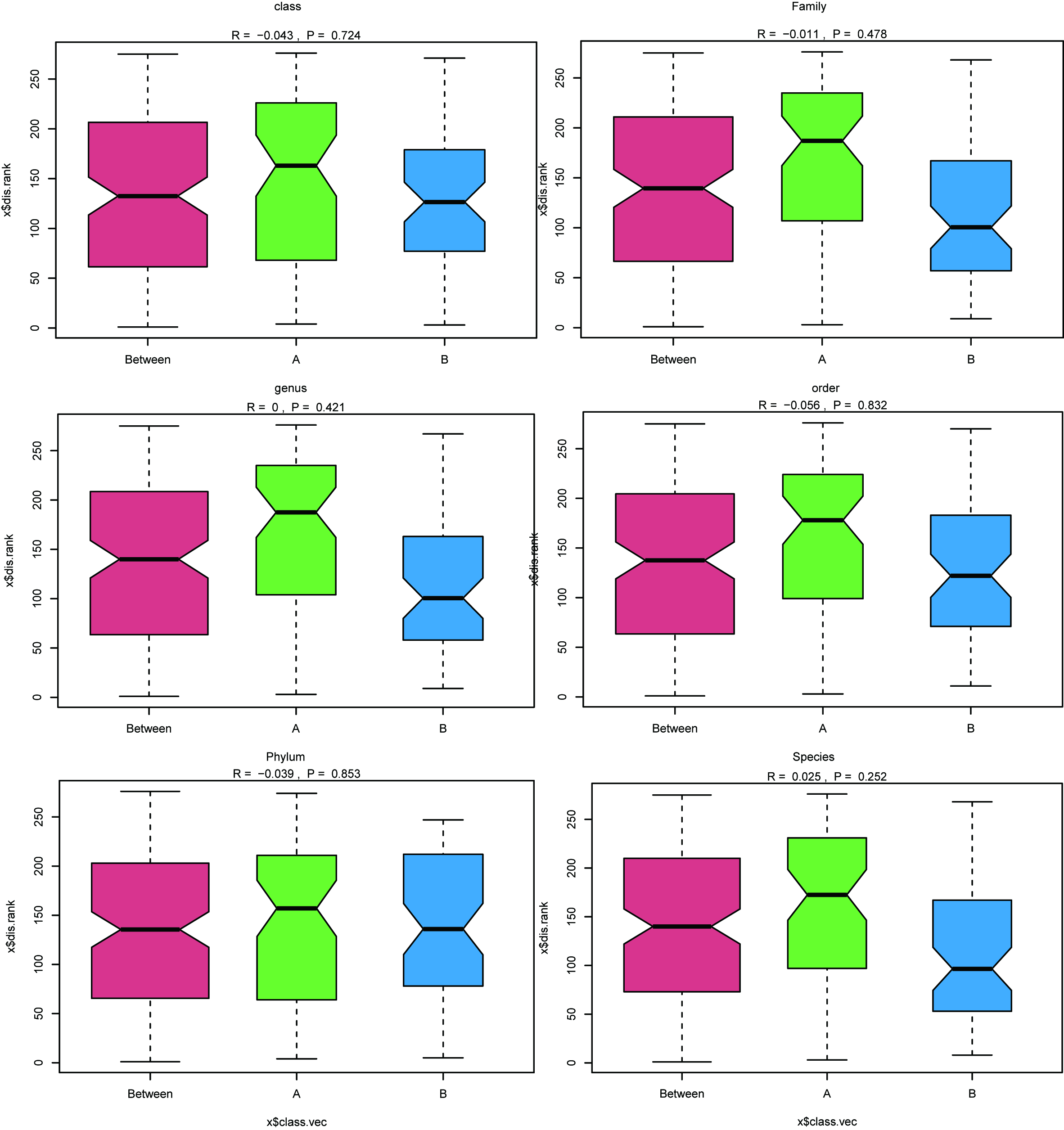

Supplement: Supplementary file 2 [file Supplementaryfile1.zip › Supplement Figure1-7/Supplement_Figure_1_bacterial.beta_diversity.tif]

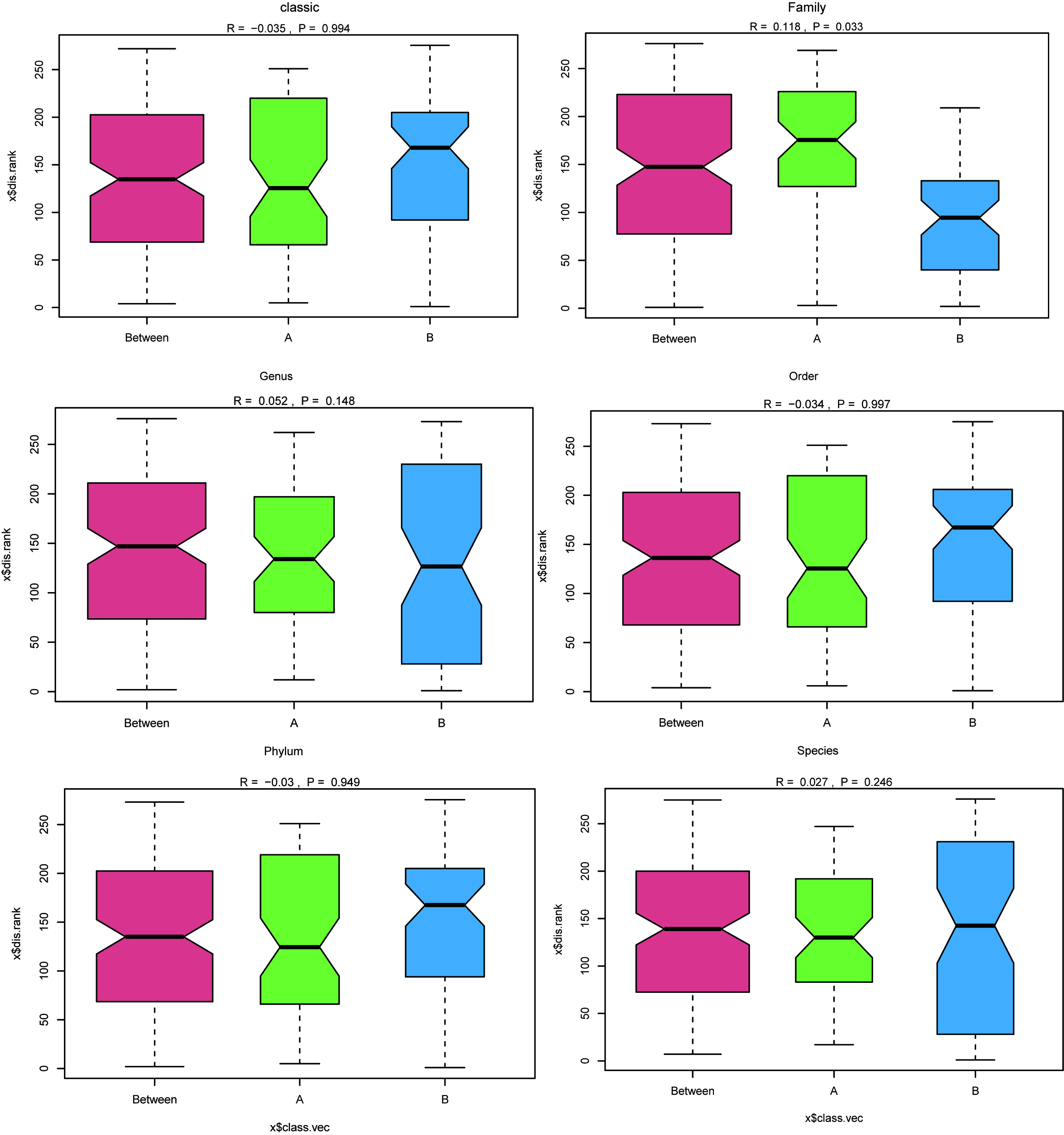

Supplement: Supplementary file 2 [file Supplementaryfile1.zip › Supplement Figure1-7/Supplement_Figure_2_virusl.beta_diversity.tif]

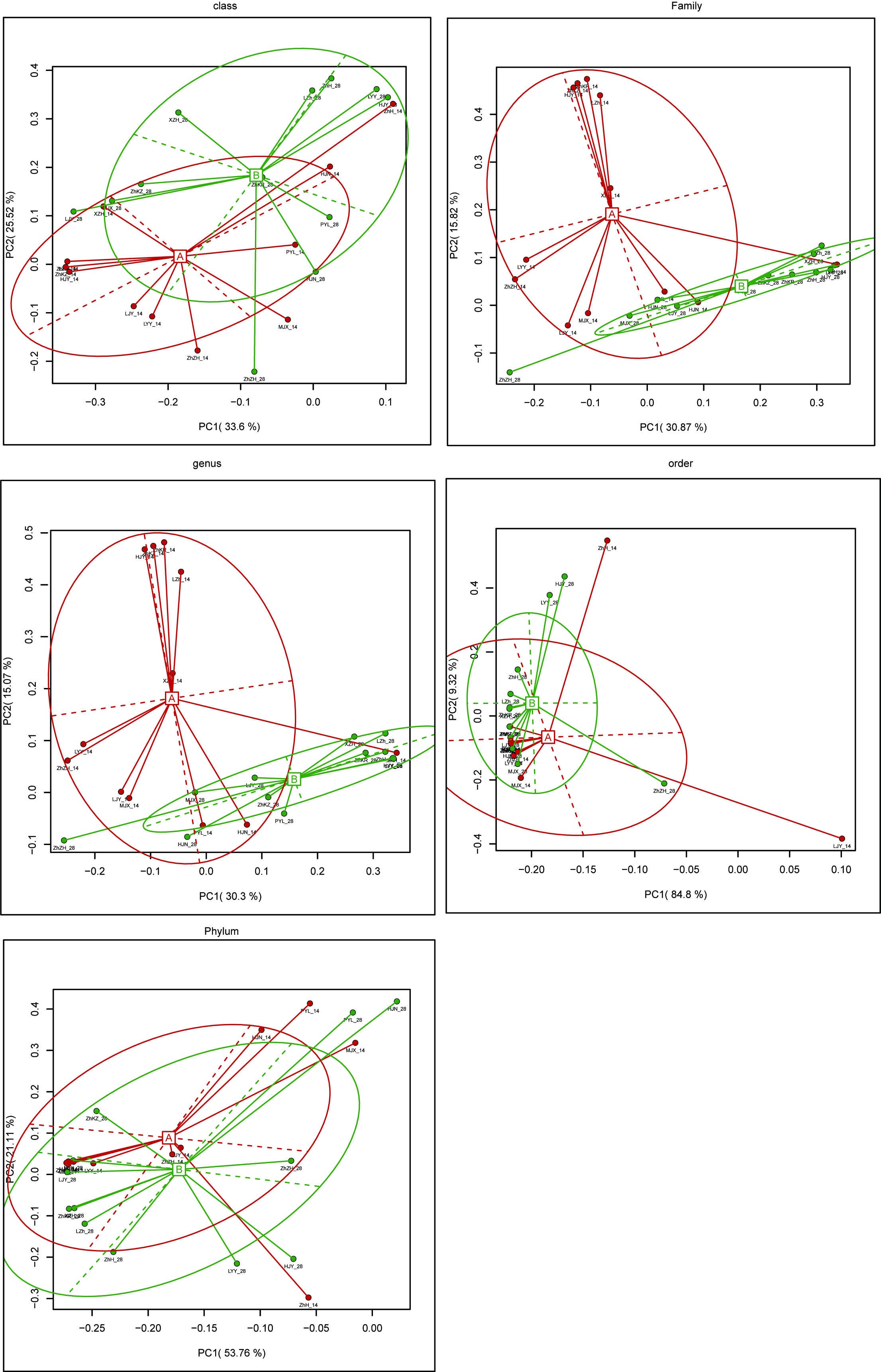

Supplement: Supplementary file 2 [file Supplementaryfile1.zip › Supplement Figure1-7/Supplement_Figure_3_bacterial.PCA.TaxLevels.tif]

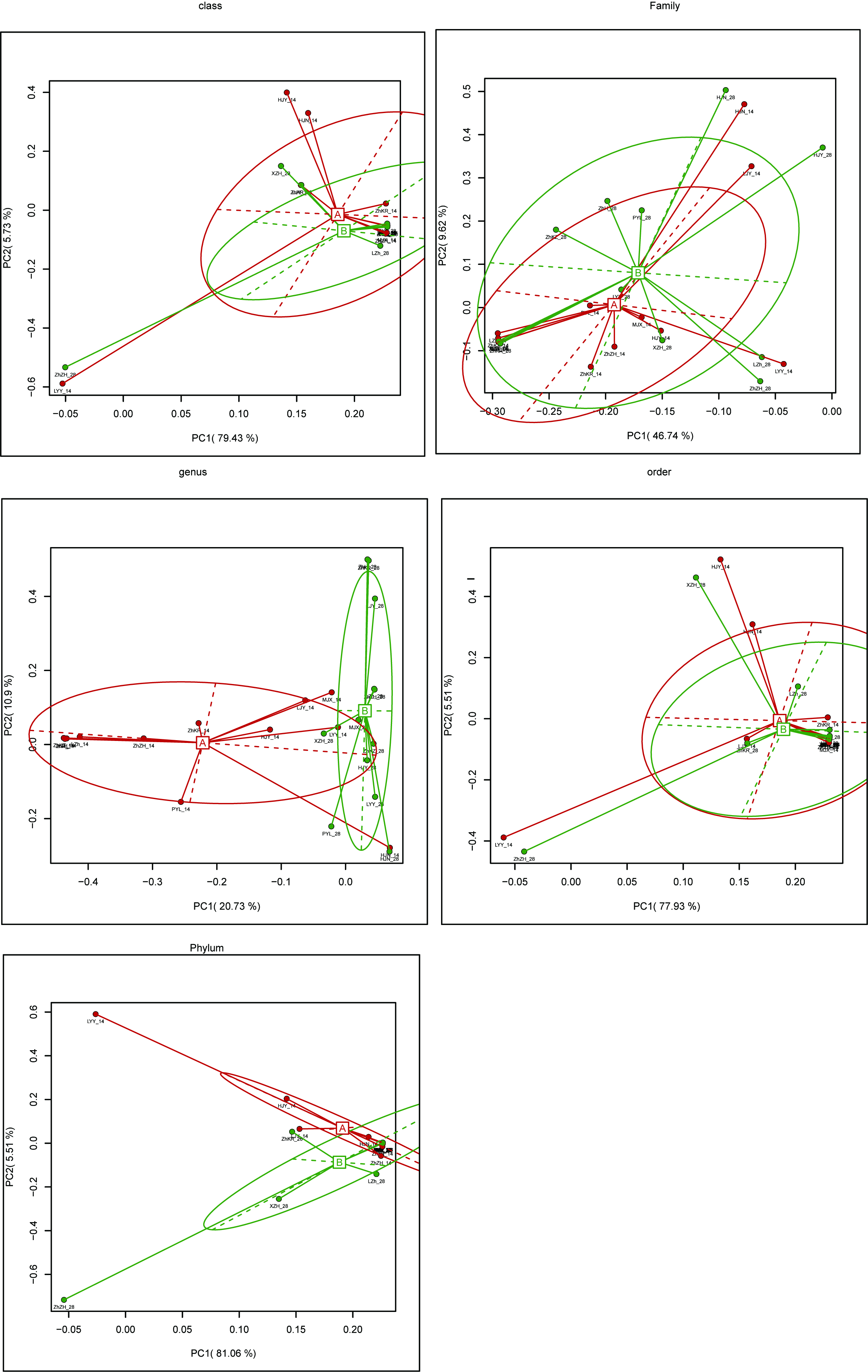

Supplement: Supplementary file 2 [file Supplementaryfile1.zip › Supplement Figure1-7/Supplement_Figure_4_virus.PCA.TaxLevels.tif]

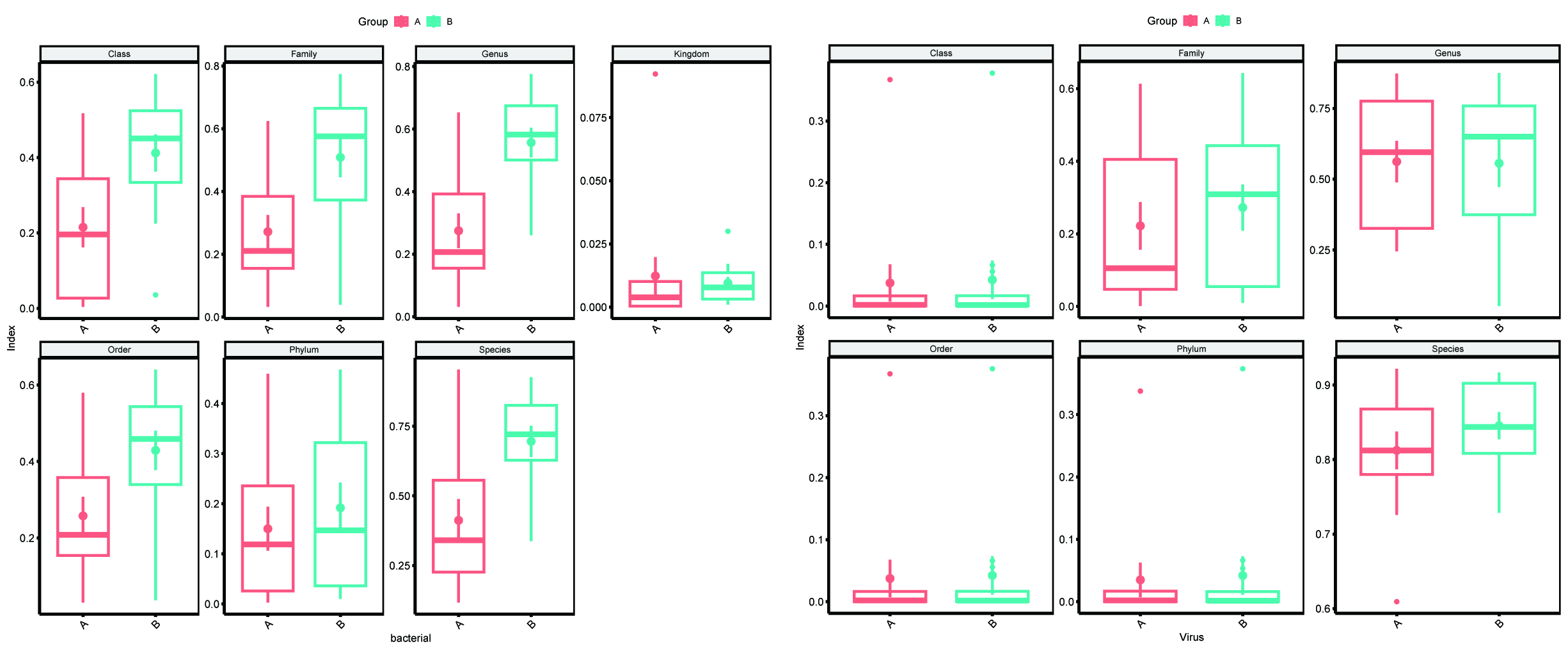

Supplement: Supplementary file 2 [file Supplementaryfile1.zip › Supplement Figure1-7/Supplement_Figure_5_alpha_diversity_simpson.tif]

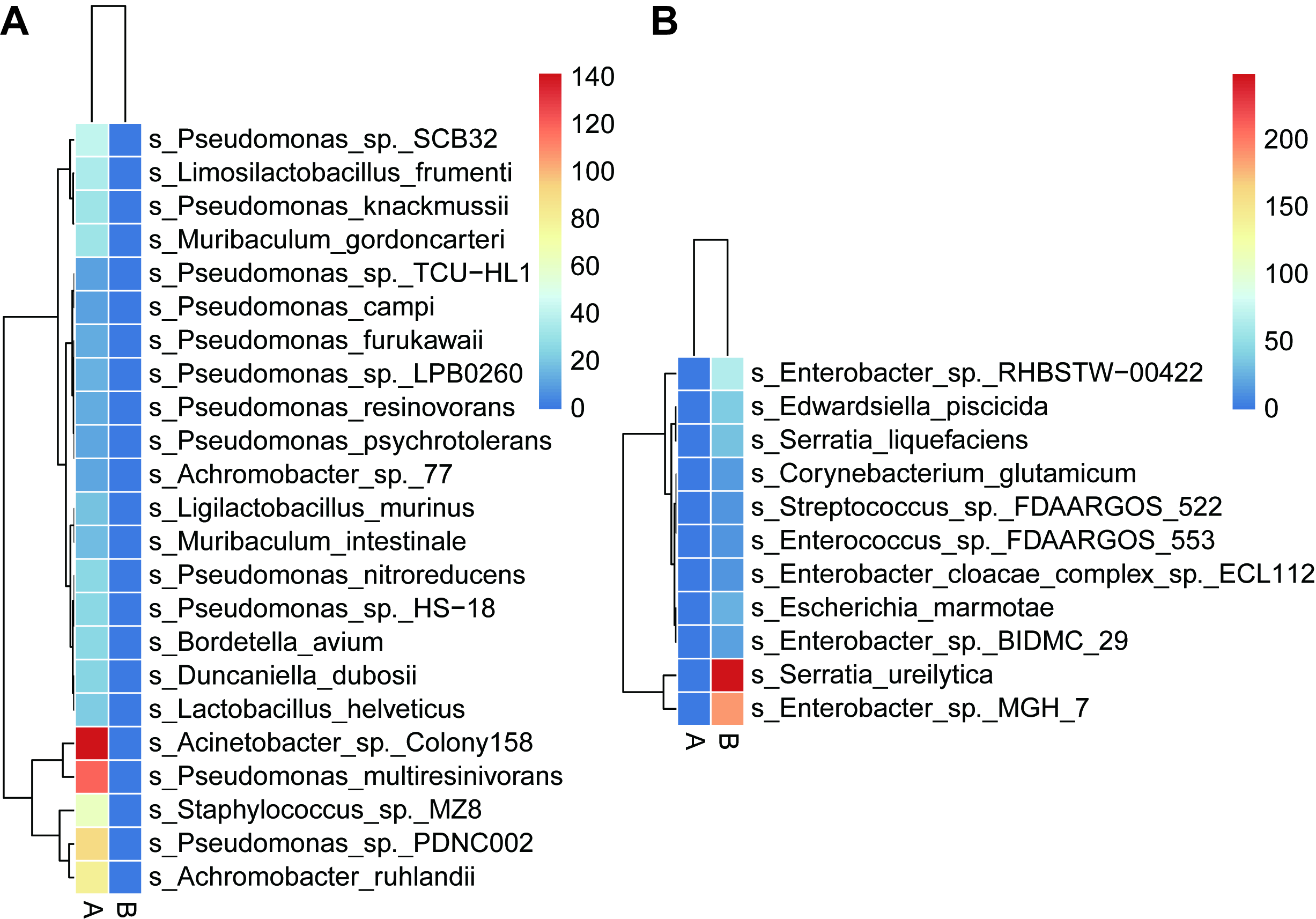

Supplement: Supplementary file 2 [file Supplementaryfile1.zip › Supplement Figure1-7/Supplement_Figure_6_Bacterial.Group.uniq.tif]

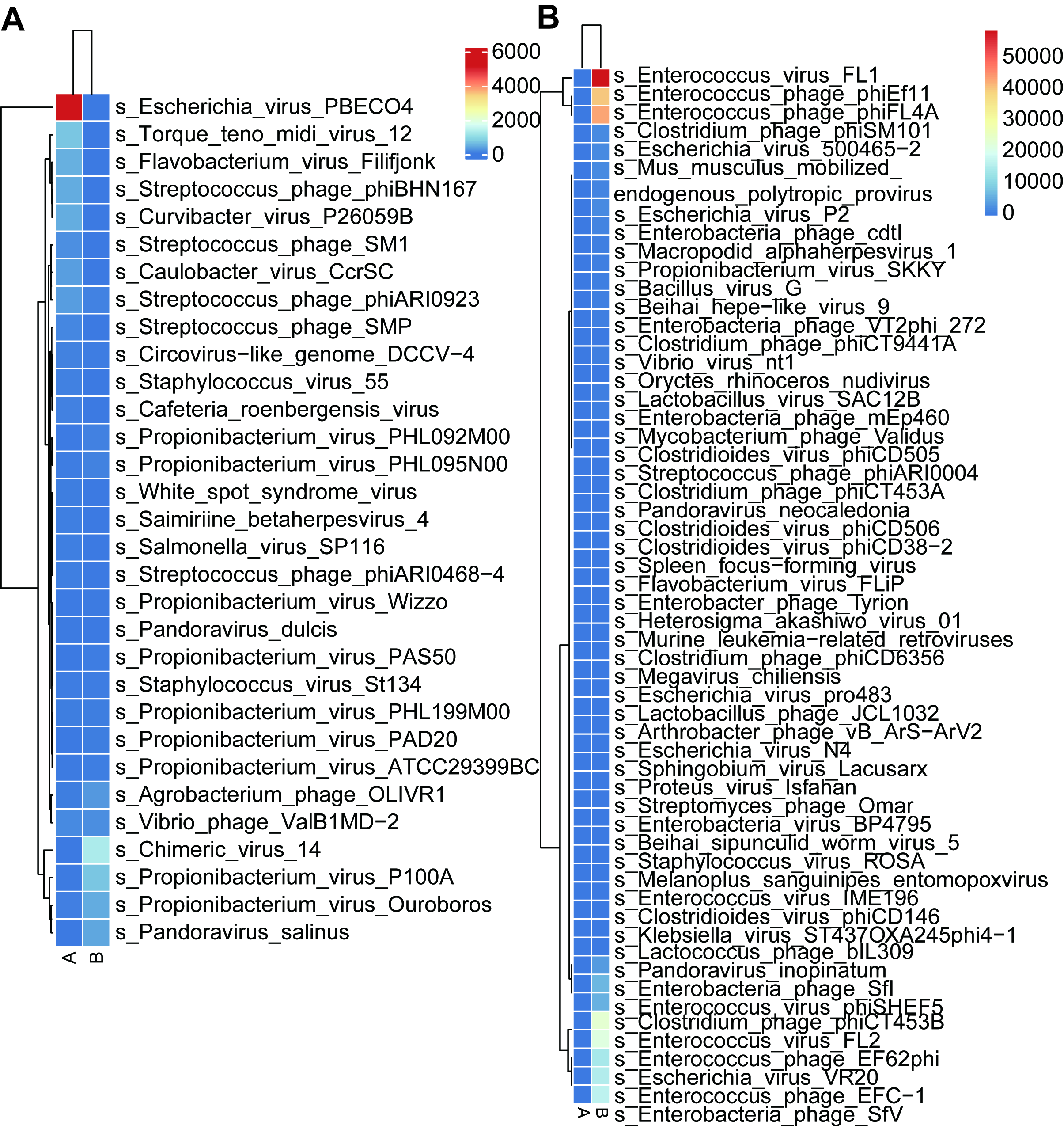

Supplement: Supplementary file 2 [file Supplementaryfile1.zip › Supplement Figure1-7/Supplement_Figure_7_Virual.Group.uniq.tif]
